# Porcine reproductive and respiratory syndrome virus triggers Golgi apparatus fragmentation-mediated autophagy to facilitate viral self-replication

**DOI:** 10.1128/jvi.01842-23

**Published:** 2024-01-05

**Authors:** Shuang-shuang Zhao, Qisheng Qian, Xin-xin Chen, Qingxia Lu, Guangxu Xing, Songlin Qiao, Rui Li, Gaiping Zhang

**Affiliations:** 1 College of Veterinary Medicine, Jilin University, Changchun, Jilin, China; 2 Key Laboratory of Animal Immunology of the Ministry of Agriculture, Henan Provincial Key Laboratory of Animal Immunology, Henan Academy of Agricultural Sciences, Zhengzhou, Henan, China; 3 Longhu Modern Immunology Laboratory, Zhengzhou, Henan, China; University of Michigan Medical School, Ann Arbor, Michigan, USA

**Keywords:** autophagy, GA fragmentation, GRASP65, Nsp2, PRRSV, RAB2, ULK1

## Abstract

Macroautophagy/autophagy is a cellular degradation and recycling process that maintains the homeostasis of organisms. A growing number of studies have reported that autophagy participates in infection by a variety of viruses. Porcine reproductive and respiratory syndrome virus (PRRSV) causes severe financial losses to the global swine industry. Although much research has shown that PRRSV triggers autophagy for its own benefits, the exact molecular mechanisms involved in PRRSV-triggered autophagy remain to be fully elucidated. In the current study, we demonstrated that PRRSV infection significantly induced Golgi apparatus (GA) fragmentation, which promoted autophagy to facilitate viral self-replication. Mechanistically, PRRSV nonstructural protein 2 was identified to interact with and degrade the Golgi reassembly and stacking protein 65 dependent on its papain-like cysteine protease 2 activity, resulting in GA fragmentation. Upon GA fragmentation, GA-resident Ras-like protein in brain 2 was disassociated from Golgi matrix protein 130 and subsequently bound to unc-51 like autophagy activating kinase 1 (ULK1), which enhanced phosphorylation of ULK1 and promoted autophagy. Taken together, all these results expand the knowledge of PRRSV-triggered autophagy as well as PRRSV pathogenesis to support novel potential avenues for prevention and control of the virus. More importantly, these results provide the detailed mechanism of GA fragmentation-mediated autophagy, deepening the understanding of autophagic processes.

**IMPORTANCE:**

Porcine reproductive and respiratory syndrome virus (PRRSV) infection results in a serious swine disease affecting pig farming worldwide. Despite that numerous studies have shown that PRRSV triggers autophagy for its self-replication, how PRRSV induces autophagy is incompletely understood. Here, we identify that PRRSV Nsp2 degrades GRASP65 to induce GA fragmentation, which dissociates RAB2 from GM130 and activates RAB2-ULK1-mediated autophagy to enhance viral replication. This work expands our understanding of PRRSV-induced autophagy and PRRSV replication, which is beneficial for anti-viral drug development.

## INTRODUCTION

In eukaryotic cells, macroautophagy/autophagy is an evolutionarily conserved mechanism that transports long-lived cytoplasmic proteins and damaged organelles to lysosomes for degradation, thereby maintaining cellular homeostasis (1, 2). In addition, autophagy is a potent antiviral defense response by which host cells recognize and degrade viral components (3–5). However, viruses have evolved equally elegant strategies to circumvent autophagic degradation, and growing evidences prove that a variety of viruses exploit autophagy to replicate efficiently within the infected cells (4, 6).

Porcine reproductive and respiratory syndrome (PRRS) is a highly infectious disease characterized by reproductive disorders in sows of late-term gestation and respiratory diseases in pigs of all ages (7). PRRS has caused tremendous economic losses in the global swine industry (8). PRRS causative agent, PRRS virus (PRRSV), is an enveloped, nonsegmented, single-stranded positive-sense RNA virus belonging to the order *Nidovirales*, the family *Arteriviridae*, and the genus *Betaarterivirus* (9). The PRRSV genome is approximately 15.4 kb in size and encodes nonstructural proteins (Nsps), including Nsp1α, Nsp1β, and Nsp2-12 (10) and structural proteins [glycoprotein (GP) 2, envelope protein, GP3, GP4, GP5, GP5a, matrix protein, and nucleocapsid (N) protein] (11, 12). PRRSV infects host cells, such as porcine alveolar macrophages (PAMs) (13), African green monkey kidney epithelial cell line MA-104, and its derivative MARC-145 (14), and utilizes the intracellular machinery to complete its replication cycle.

Previous studies have reported that PRRSV induces autophagy to facilitate its proliferation (15–17). Later studies have further shown that PRRSV Nsps, including Nsp2, Nsp3, and Nsp5, play critical roles in the induction of autophagy (18–21). For example, PRRSV Nsp2 is determined to trigger autophagy through induction of endoplasmic reticulum stress or aggresome formation (19, 20). Despite these numerous studies, the detailed mechanisms by which PRRSV triggers autophagy remain incompletely elucidated.

Golgi apparatus (GA) is an organelle functioning in protein processing, modification, sorting, and transport (22, 23). Therefore, GA is involved in various biological processes, such as lipid metabolism, ion homeostasis, stress response, and immune response (24, 25). GA is composed of a series of flattened membrane sacs and vesicular stacks, which localize in a perinuclear region (26). Under certain physiological and pathological circumstances, GA disorganizes into membrane fragments and small vesicles and diffuses throughout the cytoplasm, namely, GA fragmentation (27). GA fragmentation has been revealed to be involved in autophagy (28).

In this study, we observed that PRRSV infection induced GA fragmentation through confocal microscopy and transmission electron microscopy (TEM). Subsequently, we identified the specific PRRSV protein(s) responsible for GA fragmentation and explored how the identified PRRSV protein(s) induced GA fragmentation. Next, we determined that PRRSV-induced GA fragmentation promoted autophagy, which facilitated the viral replication.

## RESULTS

### PRRSV infection induces GA fragmentation

**Fig 1:**
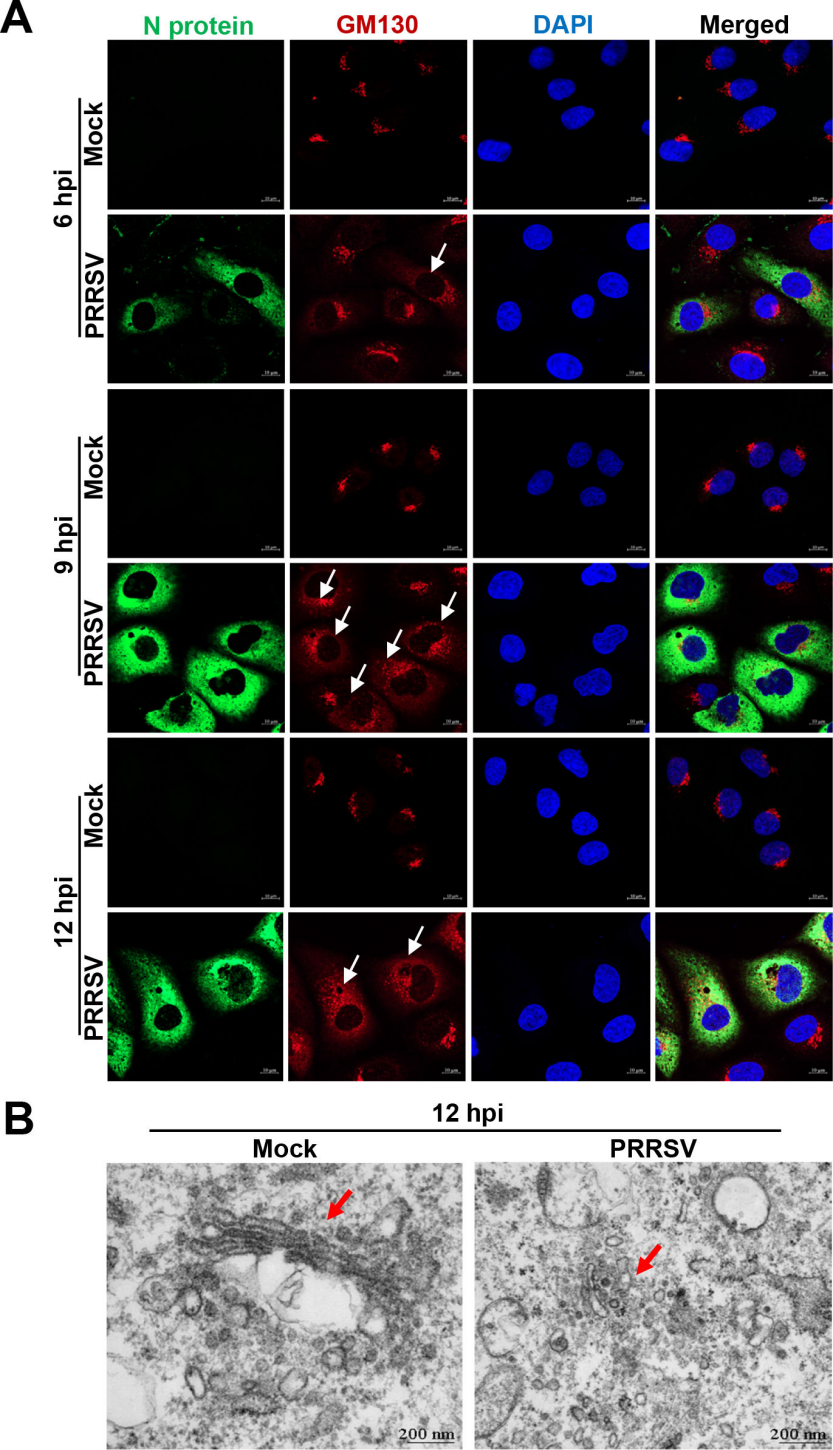
PRRSV infection induces GA fragmentation. (**A**) MARC-145 cells were mock infected (DMEM containing 2% FBS) or infected with PRRSV strain HN07-1 at an multiplicity of infection (MOI) of 1, and collected at 6, 9, and 12 hpi. GM130 and PRRSV N proteins were visualized with the specific primary and secondary antibodies. Cell nuclei were stained with DAPI. The fluorescent signals were observed with confocal microscopy (scale bars = 10 µm). (**B**) The mock-infected and PRRSV-infected (MOI = 10) MARC-145 cells were collected at 12 hpi. GA morphology was detected by TEM (scale bars = 200 nm). White/red arrows indicated GA morphology.

Firstly, we monitored the dynamic changes of GA morphology in the MARC-145 cells infected with or without the highly pathogenic (HP)-PRRSV strain HN07-1 via confocal microscopy. Golgi matrix protein 130 (GM130) and N protein were visualized as hallmarks for GA morphology and PRRSV infection, respectively. As shown in Fig. 1A, GA was located adjacent to the nuclei with a continuous network of tubular structures in the mock-infected [Dulbecco modified Eagle medium (DMEM) containing 2% fetal bovine serum (FBS)] and un-infected (inoculated with PRRSV but not infected by the virus yet) cells. In contrast, GA began to disorganize into membrane fragments and small vesicles scattered in the cytoplasm of certain PRRSV-infected cells at 6 h post infection (hpi). Obvious GA fragmentation was observed at 9 hpi, and complete GA fragmentation was found at 12, 24, and 36 hpi in the PRRSV-infected cells (Fig. 1A; Fig. S1A). To further corroborate the effect of PRRSV infection on the GA structure, we examined GA morphology in the mock-infected or PRRSV-infected MARC-145 cells at 12 hpi using TEM. Consistent with the confocal microscopy results, PRRSV infection disrupted the flattened membrane sacs and vesicular stacks of GA (Fig. 1B). To determine whether PRRSV infection induced GA fragmentation in a strain-dependent manner, we applied another two PRRSV strains, low pathogenic BJ-4 and NADC30-like HNhx. We identified that both these two PRRSV strains induced GA fragmentation in MARC-145 cells (Fig. S1B and C). As PAMs are the primary *in vivo* target cells for PRRSV (13), we tested whether PRRSV infection induced GA fragmentation in CRL-2843-CD163 cells, the immortalized PAMs stably expressing the indispensable receptor CD163 for PRRSV (29, 30). The results showed that PRRSV infection also induced GA fragmentation in CRL-2843-CD163 cells (Fig. S1D), indicating that PRRSV-induced GA fragmentation was generic in different host cells. These data provide evidence that PRRSV infection induces GA fragmentation.

### PRRSV Nsp2 induces GA fragmentation

**Fig 2:**
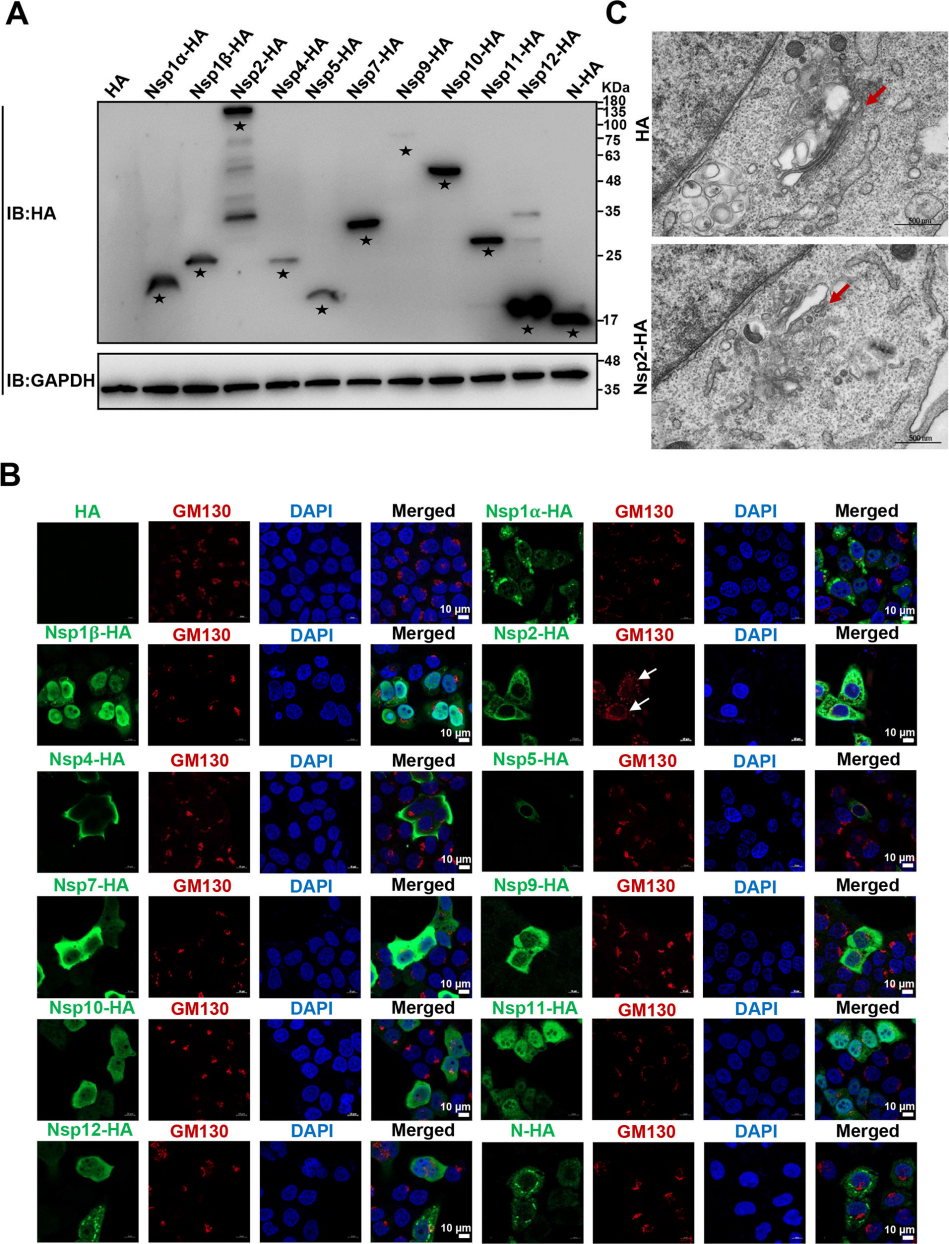
PRRSV Nsp2 induces GA fragmentation. (**A**) HeLa cells were transfected with the plasmids encoding HA-tagged PRRSV strain HN07-1 proteins or HA-tagged empty vector, followed by IB analysis with anti-HA antibody. Asterisks marked the target proteins. (**B**) HeLa cells were overexpressed with HA-tagged PRRSV strain HN07-1 proteins. GM130 and HA-tagged PRRSV proteins were visualized with the specific primary and secondary antibodies. Cell nuclei were stained with DAPI. GA morphology was assessed by confocal microscopy (scale bars = 10 µm). (**C**) HeLa cells were transfected with the Nsp2-HA plasmid or HA-tagged empty vector. GA morphology was detected by TEM (scale bars = 500 nm). White/red arrows indicated GA morphology.

To identify which PRRSV protein(s) induced GA fragmentation, we overexpressed each hemagglutinin (HA)-tagged PRRSV strain HN07-1 protein in HeLa cells and detected their expression via immunoblotting (IB). Nsp1α-HA, Nsp1β-HA, Nsp2-HA, Nsp4-HA, Nsp5-HA, Nsp7-HA, Nsp9-12-HA, and N-HA were successfully expressed (Fig. 2A). In the meantime, we performed confocal microscopy to monitor GA morphology. As shown in Fig. 2B, Nsp2-HA was identified to induce the most significant GA fragmentation. Additionally, we overexpressed Nsp2-HA in HeLa cells and observed GA fragmentation using TEM (Fig. 2C). We further validated that Nsp2-HA from PRRSV strains BJ-4 and HNhx induced significant GA fragmentation (Fig. S2). These results demonstrate that PRRSV Nsp2 induces GA fragmentation.

### PRRSV Nsp2 interacts with GRASP65

We subsequently screened the host cellular protein(s) targeted by PRRSV Nsp2 to induce GA fragmentation using immunoprecipitation (IP) along with liquid chromatography and tandem mass spectrometry (LC-MS/MS). The plasmids encoding Nsp2-HA and HA-tagged empty vector were separately transfected into the human embryonic kidney 293T cell line (HEK-293T), and IP was performed using anti-HA magnetic beads to capture endogenous host cellular proteins. Silver staining indicated immunoprecipitated protein bands, with the black arrow marking the most distinctly different one in the Nsp2-HA-overexpressed cells (~65 kDa; Fig. S3A). This band was examined by LC-MS/MS, and the most prominent Nsp2-associated protein was identified as Golgi reassembly and stacking protein 65 (GRASP65), which plays a crucial role in maintaining GA structure (31, 32).

**Fig 3:**
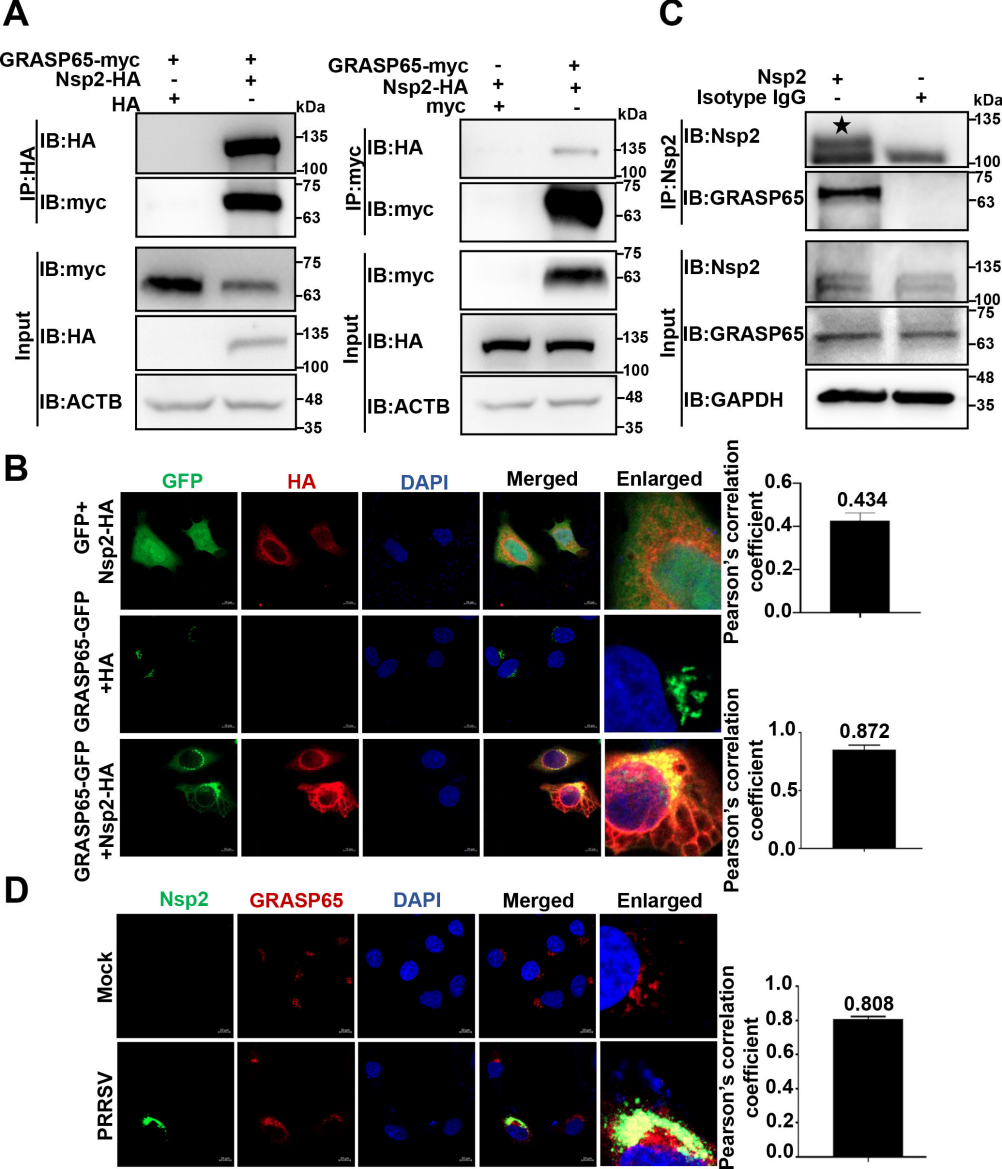
PRRSV Nsp2 interacts with GRASP65. (**A**) HEK-293T cells were transfected with the plasmids encoding Nsp2-HA and GRASP65-myc or HA/myc-tagged empty vector for 36 h, followed by co-IP with anti-HA or anti-myc magnetic beads and IB analysis with anti-HA and anti-myc antibodies. (**B**) HeLa cells were transfected with the plasmids encoding GRASP65-GFP and Nsp2-HA for 24 h. In parallel, HeLa cells were transfected with the plasmid encoding GRASP65-GFP or Nsp2-HA alone as a control. Nsp2-HA was visualized with the specific primary and secondary antibodies. Cell nuclei were stained with DAPI. The fluorescent signals were observed with confocal microscopy (scale bars = 10 µm). The co-localization was assessed by determination of the Pearson’s correlation coefficient using the JaCoP plugin in ImageJ software. (**C**) MARC-145 cells were infected with PRRSV strain HN07-1 at 0.1 MOI for 24 h. They were then analyzed via endogenous IP using protein A/G magnetic beads pre-incubated with anti-Nsp2 pAbs and IB with anti-Nsp2 and anti-GRASP65 antibodies. Asterisk marked PRRSV Nsp2. (**D**) MARC-145 cells were infected with PRRSV strain HN07-1 at 0.1 MOI for 24 h. The fluorescent signals were observed with confocal microscopy (scale bars = 10 µm). The co-localization was assessed by determination of the Pearson’s correlation coefficient using the JaCoP plugin in ImageJ software.

To confirm that Nsp2 interacted with GRASP65, we co-transfected the Nsp2-HA and GRASP65-myc plasmids in HEK-293T cells and detected an interaction between exogenous Nsp2 and GRASP65 (Fig. 3A). Meanwhile, we overexpressed Nsp2-HA and GRASP65-GFP (green fluorescent protein) in HeLa cells and observed their co-localization via confocal microscopy. Their co-localization was quantified and expressed as Pearson’s correlation coefficient. The mean value was 0.872, indicating an interaction between these two proteins (Fig. 3B). In addition, we conducted IP in the PRRSV strain HN07-1-infected MARC-145 cells and monitored endogenous Nsp2 interacted with GRASP65 (Fig. 3C). Moreover, we performed confocal microscopy and found that Nsp2 interacted with GRASP65 in the PRRSV-infected MARC-145 cells (the Pearson’s correlation coefficient was 0.808; Fig. 3D). We also validated that Nsp2 interacted with GRASP65 in the PRRSV strains BJ-4 and HNhx-infected MARC-145 cells through IP (Fig. S3B). These results substantiate that PRRSV Nsp2 specifically interacts with GRASP65.

**Fig 4:**
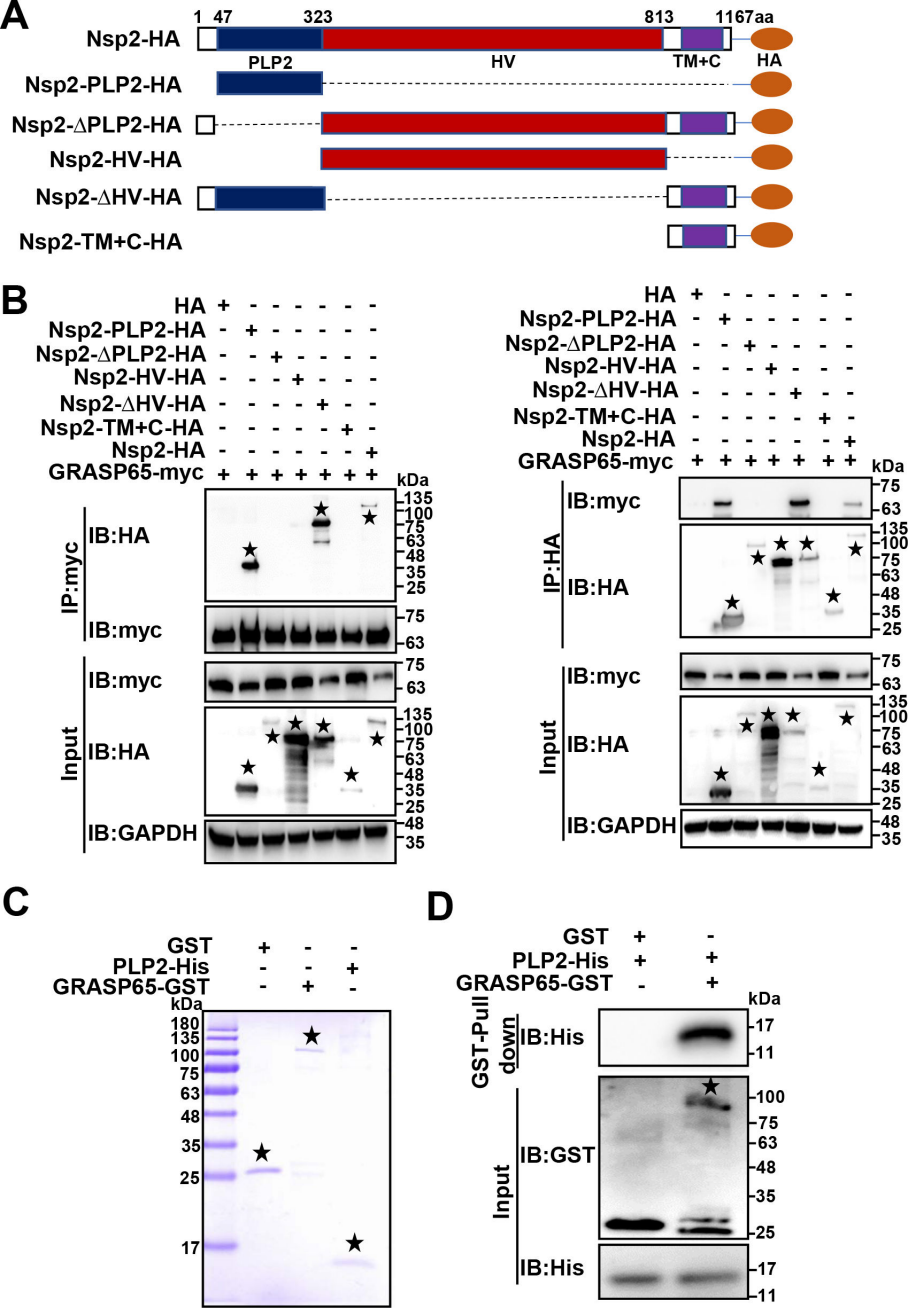
PRRSV Nsp2 PLP2 interacts with GRASP65. (**A**) The schematic diagram of HA-tagged PRRSV Nsp2 deletion mutants. (**B**) HEK-293T cells were transfected with the plasmids encoding GRASP65-myc and Nsp2-HA or the indicated Nsp2-HA deletion mutants for 36 h. They were then analyzed via co-IP with anti-myc or anti-HA magnetic beads and IB with anti-HA and anti-myc antibodies. Asterisks marked Nsp2-HA or the indicated Nsp2-HA deletion mutants. (**C**) The recombinant GRASP65-GST and PLP2-His were purified by anti-GST magnetic beads and Ni-NTA agarose, respectively, and detected by sodium dodecyl sulfate-polyacrylamide gel electrophoresis (SDS-PAGE). Asterisks marked the corresponding purified target proteins. (**D**) The purified GRASP65-GST was coupled to anti-GST magnetic beads, where GST served as a control. Then, the magnetic beads were incubated with the purified PLP2-His. The eluted samples were subjected to IB and detected by anti-His and anti-GST antibodies. Asterisk marked GRASP65-GST.

PRRSV Nsp2 is a multi-domain protein consisting of an N-terminal papain-like cysteine protease 2 (PLP2), a highly variable region (HV) in the middle, a hydrophobic transmembrane region (TM), and a C-terminal tail (C) (33, 34). To identify the region of Nsp2 involved in its interaction with GRASP65, we constructed a series of HA-tagged PRRSV Nsp2 deletion mutants and designated them as Nsp2-PLP2-HA, Nsp2-ΔPLP2-HA, Nsp2-HV-HA, Nsp2-ΔHV-HA, and Nsp2-TM+C-HA (Fig. 4A). Afterwards, we co-expressed GRASP65-myc with Nsp2-HA or the indicated Nsp2-HA deletion mutants for co-immunoprecipitation (co-IP). The results revealed that Nsp2-ΔPLP2-HA, Nsp2-HV-HA, and Nsp2-TM+C-HA did not interact with GRASP65-myc, while Nsp2-HA, Nsp2-PLP2-HA, and Nsp2-ΔHV-HA did (Fig. 4B). These data verified that PRRSV Nsp2 PLP2 interacted with GRASP65. To validate the direct interaction between PRRSV Nsp2 PLP2 and GRASP65, we conducted a glutathione S-transferase (GST) pulldown assay. Recombinant GRASP65-GST and PLP2-His were successfully expressed as soluble proteins and purified to high purity (Fig. 4C). We utilized the purified GRASP65-GST to pull down the PLP2-His, with GST as a control. The results showed that PLP2-His specially bound to GRASP65-GST (Fig. 4D). These data indicate that PRRSV Nsp2 PLP2 directly interacts with GRASP65.

### PRRSV Nsp2 induces GA fragmentation by degrading GRASP65

**Fig 5:**
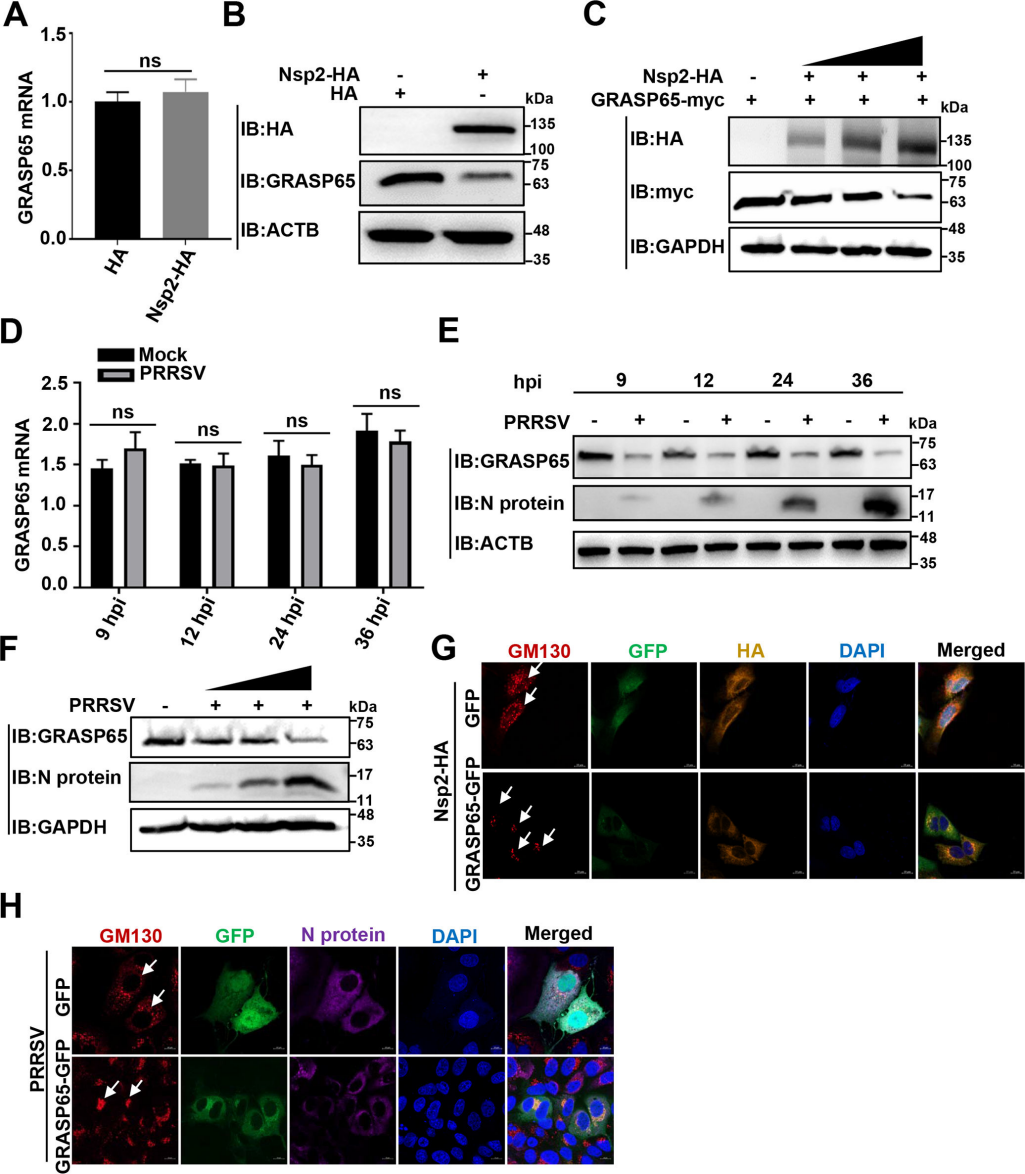
PRRSV Nsp2 induces GA fragmentation by degrading GRASP65. (**A and B**) HEK-293T cells were transfected with the plasmid encoding Nsp2-HA or HA-tagged empty vector (1.5 µg) for 24 h. (**A**) The relative GRASP65 mRNA abundance was analyzed using quantitative real-time polymerase chain reaction (RT-qPCR). (**B**) Endogenous GRASP65 protein level was detected by IB. (**C**) Gradient concentrations of the Nsp2-HA plasmid (0.5 µg, 1 µg, and 1.5 µg) were co-transfected with the GRASP65-myc plasmid into HEK-293T cells for 24 h. GRASP65 protein level was detected by IB. (**D and E**) MARC-145 cells were infected with PRRSV (MOI = 0.1) or mock infected and collected at the indicated time points (9 hpi, 12 hpi, 24 hpi, and 36 hpi). (**D**) The relative GRASP65 mRNA abundance was analyzed using RT-qPCR. (**E**) Endogenous GRASP65 protein abundance was detected by IB. (**F**) MARC-145 cells were infected with PRRSV at different MOIs (0.5 MOI, 1 MOI, and 5 MOI) or mock infected and collected at 24 hpi. Endogenous GRASP65 protein level was detected by IB. (**G**) HeLa cells were transfected with the plasmid encoding GRASP65-GFP or GFP-tagged empty vector for 24 h and then transfected with the plasmid encoding Nsp2-HA for 24 h. GM130 and Nsp2-HA were visualized with the specific primary and secondary antibodies. Cell nuclei were stained with DAPI. The morphology of GA was assessed by confocal microscopy (scale bars = 10 µm). (**H**) MARC-145 cells were transfected with the plasmid encoding GRASP65-GFP or GFP-tagged empty vector for 24 h and then infected with PRRSV (1 MOI) for 12 h. GM130 and N proteins were visualized with the specific primary and secondary antibodies. Cell nuclei were stained with DAPI. The morphology of GA was assessed by confocal microscopy (scale bars = 10 µm). Statistical analysis was carried out using Student’s *t* test. ns, not significant (*P* > 0.05). White arrows indicated GA morphology.

The protein level of GRASP65 is critical for sustaining GA morphology (32). Since GRASP65 was targeted by PRRSV Nsp2 as described above, we hypothesized that PRRSV Nsp2 induced GA fragmentation by degrading GRASP65. To prove our hypothesis, we investigated the effect of Nsp2 overexpression on the RNA and protein levels of endogenous GRASP65. As shown in Fig. 5A and B, the RNA level of GRASP65 was stable, whereas the protein level of GRASP65 was significantly lowered by Nsp2-HA overexpression. Furthermore, we found that overexpression of Nsp2 decreased exogenous GRASP65 in a dose-dependent manner (Fig. 5C). Moreover, we detected the stable RNA abundance but lowered protein abundance of endogenous GRASP65 in the PRRSV-infected MARC-145 cells at different time points (Fig. 5D and E). Additionally, the protein level of GRASP65 was declined by PRRSV infection in a dose-dependent manner (Fig. 5F). All of these results show that GRASP65 is degraded by PRRSV Nsp2.

Considering this fact, we speculated that GRASP65 overexpression probably antagonized Nsp2-induced GA fragmentation. As shown in Fig. 5G, GA fragmentation was observed in the Nsp2-HA and GFP-co-expressed cells. However, GRASP65-GFP overexpression kept GA morphology in the Nsp2-HA-overexpressed cells (Fig. 5G). Similarly, GA fragmentation was antagonized during PRRSV infection in the GRASP65-GFP-overexpressed cells (Fig. 5H). In summary, these data illuminate that PRRSV Nsp2 induces GA fragmentation by degrading GRASP65.

### The cysteine protease activity of PRRSV Nsp2 is responsible for degrading GRASP65 to induce GA fragmentation

**Fig 6:**
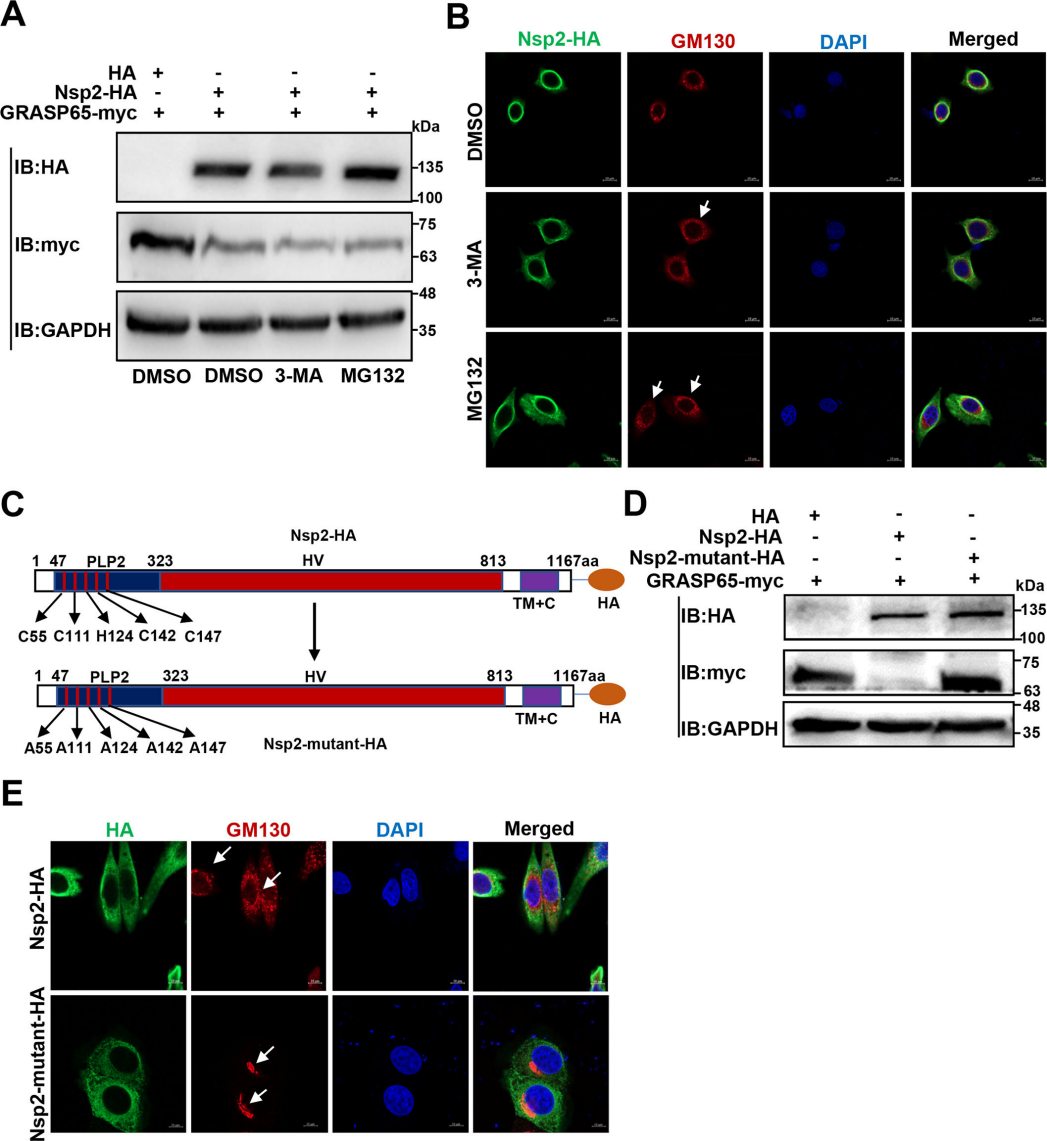
PRRSV Nsp2 induces GA fragmentation by degrading GRASP65 dependent on its cysteine protease activity. (**A**) HEK-293T cells were transfected with the plasmids encoding GRASP65-myc and Nsp2-HA or HA-tagged empty vector. The inhibitors 3-MA and MG132 or DMSO were added 12 h before the collection of the samples. GRASP65 protein abundance was detected by IB. (**B**) HeLa cells were transfected with the plasmid encoding Nsp2-HA for 6 h. The inhibitors 3-MA, MG132, or DMSO were added, and the samples were collected after 24 h. GM130 and Nsp2-HA were visualized with the specific primary and secondary antibodies. Cell nuclei were stained with DAPI. The morphology of GA was assessed by confocal microscopy (scale bars = 10 µm). (**C**) The schematic diagram of Nsp2-mutant-HA. (**D**) HEK-293T cells were co-transfected with the plasmids encoding GRASP65-myc and Nsp2-HA or Nsp2-mutant-HA. At 24 h post-transfection, the cell lysates were collected for analysis of the GRASP65 protein level with IB. (**E**) HeLa cells were transfected with the plasmid encoding Nsp2-HA or Nsp2-mutant-HA for 24 h. GM130, Nsp2-HA, and Nsp2-mutant-HA were visualized with the specific primary and secondary antibodies. Cell nuclei were stained with DAPI. The morphology of GA was assessed by confocal microscopy (scale bars = 10 µm). White arrows indicated GA morphology.

We explored how PRRSV Nsp2 degrades GRASP65 to induce GA fragmentation. As the autolysosomal pathway and ubiquitin-proteasome system are two main host cellular degradation routes (35, 36), we initially examined whether PRRSV Nsp2 degraded GRASP65 via these two routes using the autophagy inhibitor 3-methyladenine (3-MA) and the proteasome inhibitor MG132, respectively (37, 38). As shown in Fig. 6A and B, these two inhibitors neither restored the protein level of GRASP65 nor recovered GA structure in the Nsp2-HA-overexpressed cells. Therefore, we conclude that Nsp2 itself plays a crucial role in the degradation of GRASP65 to induce GA fragmentation.

PRRSV Nsp2 possesses cysteine protease activity, where C55, C111, H124, C142, and C147 are highly conserved and responsible for the activity (39). We mutated these residues to alanine (named as Nsp2-mutant-HA; Fig. 6C) and detected that Nsp2-mutant-HA lost the ability to degrade GRASP65 (Fig. 6D) and failed to induce GA fragmentation (Fig. 6E). These data illustrate that the cysteine protease activity of PRRSV Nsp2 is responsible for degrading GRASP65 to induce GA fragmentation.

### PRRSV Nsp2 induces GA fragmentation to promote autophagy

**Fig 7:**
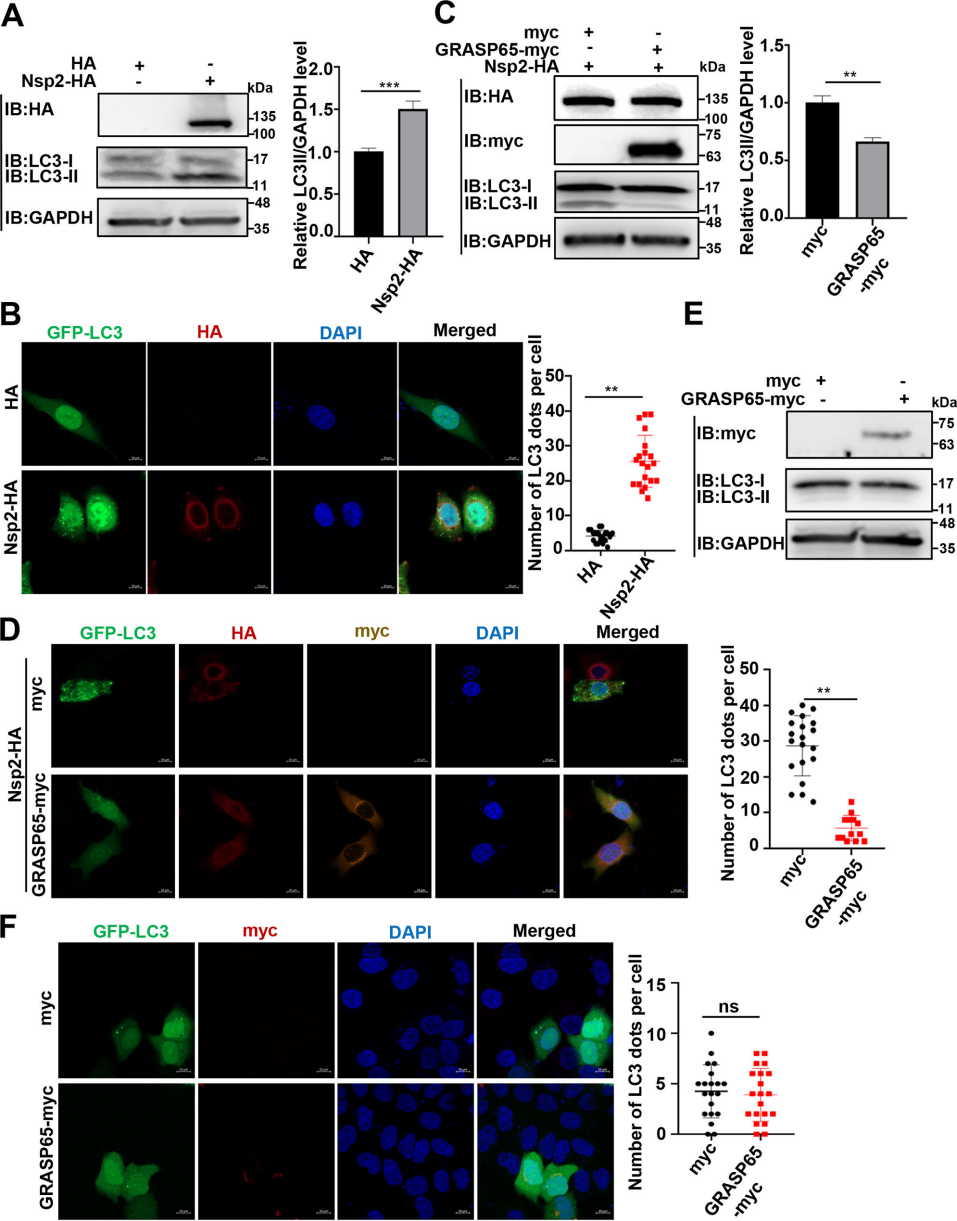
GRASP65 overexpression antagonizes Nsp2-triggered autophagy. (**A**) HEK-293T cells were transfected with the plasmid encoding Nsp2-HA or HA-tagged empty vector. LC3-I and LC3-II were assessed by IB. (**B**) HeLa cells were transfected with the plasmids encoding GFP-LC3 and Nsp2-HA or HA-tagged empty vector. The fluorescence of GFP-LC3 was detected by confocal microscopy. The numbers of GFP-LC3 dots were quantified from 20 different cells in each experimental group (scale bars = 10 µm). (**C**) HEK-293T cells were transfected with the plasmids encoding Nsp2-HA and GRASP65-myc or myc-tagged empty vector. LC3-I and LC3-II were assessed by IB. (**D**) HeLa cells were transfected with the plasmids encoding GFP-LC3, Nsp2-HA, and GRASP65-myc or myc-tagged empty vector. The fluorescence of GFP-LC3 was detected by confocal microscopy. The numbers of GFP-LC3 dots were quantified from 20 different cells in each experimental group (scale bars = 10 µm). (**E**) HEK-293T cells were transfected with the plasmid encoding GRASP65-myc or myc-tagged empty vector. LC3-I and LC3-II were assessed by IB. (**F**) HeLa cells were transfected with the plasmid encoding GFP-LC3 and GRASP65-myc or myc-tagged empty vector. The fluorescence of GFP-LC3 was detected by confocal microscopy. The numbers of GFP-LC3 dots were quantified from 20 different cells in each experimental group (scale bars = 10 µm). Data represent means ± SEM from three independent experiments. Statistical analysis was carried out using Student’s *t* test. ns, not significant (*P* > 0.05); **P < 0.01 and ***P < 0.001.

Next, we investigated the biological significance of PRRSV Nsp2-induced GA fragmentation. PRRSV Nsp2 has been previously reported to trigger autophagy (19, 20). There is also a study showing that GA fragmentation is involved in autophagy (40). Therefore, we determined whether PRRSV Nsp2-induced GA fragmentation was associated with autophagy. During autophagy, microtubule-associated protein 1 light chain 3 (LC3-I) undergoes lipidation to form LC3-II (41). In addition, LC3 redistributes from a diffuse cytoplasmic localization to a distinctive punctate cytoplasmic pattern (15). As shown in Fig. 7A and B, Nsp2-HA overexpression triggered autophagy as indicated by the increased LC3-II level and punctate GFP-LC3 formation. In contrast, GRASP65-myc overexpression suppressed Nsp2-triggered autophagy as shown by a weakened band of LC3-II (Fig. 7C) and impaired punctate distribution of GFP-LC3 (Fig. 7D). As GRASP65 overexpression was shown to restore GA structure in the Nsp2-overexpressed cells (Fig. 5), these results suggest that GRASP65 overexpression antagonizes Nsp2-induced GA fragmentation and thus suppresses Nsp2-triggered autophagy. To exclude the effect of GRASP65 itself on autophagy, we transfected the GRASP65-myc plasmid alone into HEK-293T cells but did not observe significant autophagy (Fig. 7E). Similarly, we overexpressed GRASP65-myc and GFP-LC3 in HeLa cells and found that GFP-LC3 still exhibited a diffuse distribution in the cytoplasm (Fig. 7F). These data show that Nsp2 induces GA fragmentation to promote autophagy.

### PRRSV Nsp2-induced GA fragmentation disassociates RAB2 from GM130 to enhance its interaction with ULK1

**Fig 8:**
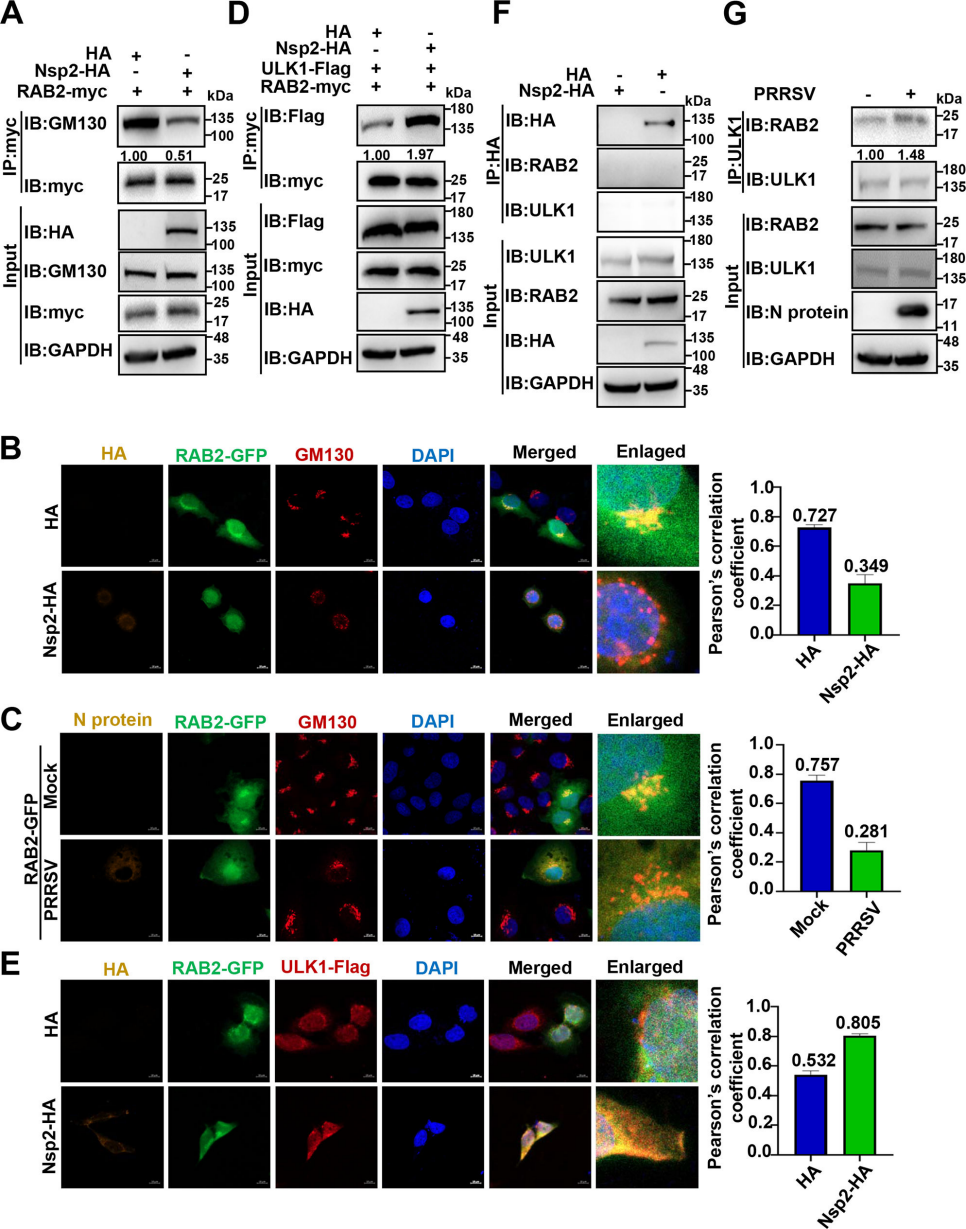
PRRSV Nsp2-induced GA fragmentation disassociates RAB2 from GM130 to enhance its interaction with ULK1. (**A and B**) HEK-293T or HeLa cells were transfected with the plasmids encoding RAB2-myc/GFP and Nsp2-HA or HA-tagged empty vector for 36 h. (**A**) Co-IP was performed with anti-myc magnetic beads, and IB was conducted with the specific antibodies. (**B**) The fluorescent signals were observed with confocal microscopy. The co-localization was assessed by determination of the Pearson’s correlation coefficient (scale bars = 10 µm). (**C**) MARC-145 cells were transfected with the plasmid encoding RAB2-GFP. At 24 h post-transfection, the MARC-145 cells were infected with PRRSV at an MOI of 1 or mock infected, and the fluorescent signals were observed with confocal microscopy. The co-localization was assessed by determination of the Pearson’s correlation coefficient (scale bars = 10 µm). (**D**) HEK-293T cells were co-transfected with the plasmids encoding RAB2-myc, ULK1-Flag, and Nsp2-HA or HA-tagged empty vector for 36 h. Co-IP was performed with anti-myc magnetic beads, and IB was conducted with the specific antibodies. (**E**) HeLa cells were co-transfected with the plasmids encoding RAB2-GFP, ULK1-Flag, and Nsp2-HA or HA-tagged empty vector for 36 h. The fluorescent signals were observed with confocal microscopy. The co-localization was assessed by determination of the Pearson’s correlation coefficient (scale bars = 10 µm). (**F**) HEK-293T cells were transfected with the plasmid encoding Nsp2-HA or HA-tagged empty vector for 36 h, followed by IP with anti-HA magnetic beads and IB analysis with the specific antibodies. (**G**) MARC-145 cells were infected with PRRSV at 0.1 MOI or mock infected for 24 h. The cell lysates were immunoprecipitated by the anti-ULK1 antibody pre-incubated with protein A/G magnetic beads. IB was conducted with the specific antibodies.

We subsequently took efforts to elaborate how Nsp2 induced GA fragmentation to promote autophagy. GA-resident Ras-like protein in brain 2 (RAB2) is a GA-resident protein, which is disassociated from GM130 and interacts with unc-51 like autophagy activating kinase 1 (ULK1) to enhance ULK1 phosphorylation and promote autophagy (42–45). We therefore assumed that Nsp2-induced GA fragmentation decreased the association of RAB2 with GM130 and increased its interaction with ULK1 to enhance ULK1 phosphorylation and promote autophagy. On the one hand, we found that overexpression of Nsp2-HA weakened the association of RAB2-myc with GM130 by co-IP (Fig. 8A). We further observed that Nsp2-HA overexpression decreased the association of RAB2-GFP with GM130 via confocal microscopy (the Pearson’s correlation coefficient decreased from 0.727 to 0.349; Fig. 8B). PRRSV infection also lowered the association of RAB2-GFP with GM130 (the Pearson’s correlation coefficient decreased from 0.757 to 0.281; Fig. 8C). These data suggest that Nsp2-induced GA fragmentation weakens the association between RAB2 and GM130.

On the other hand, we verified an increased interaction between RAB2-myc and ULK1-Flag in the Nsp2-HA-overexpressed cells as shown by co-IP (Fig. 8D) and confocal microscopy (the Pearson’s correlation coefficient increased from 0.532 to 0.805, Fig. 8E). As shown in Fig. 8F, there was no interaction between Nsp2-HA and RAB2 or ULK1, which precluded that Nsp2 directly interacted with RAB2 or ULK1 to enhance their interaction. Endogenous IP has also demonstrated an increased interaction between RAB2 and ULK1 in the PRRSV-infected cells (Fig. 8G). These results suggest that Nsp2-induced GA fragmentation enhances the interaction between RAB2 and ULK1.

In contrast, overexpression of GRASP65-myc restored the association of RAB2-GFP with GM130 as shown by co-IP (Fig. S4A) and confocal microscopy (the Pearson’s correlation coefficient increased from 0.373 to 0.751; Fig. S4B). In the meantime, overexpression of GRASP65-myc significantly weakened the interaction between ULK1-Flag and endogenous RAB2 in the Nsp2-HA-overexpressed or PRRSV-infected cells as assessed by co-IP (Fig. S4C and D). These results suggest that GRASP65 overexpression counteracts Nsp2-induced GA fragmentation to restore the association of RAB2 with GM130, which in turn attenuates its interaction with ULK1.

In conclusion, these data demonstrate that Nsp2-induced GA fragmentation disassociates RAB2 from GM130 to enhance its interaction with ULK1.

### PRRSV Nsp2-induced GA fragmentation activates the RAB2-ULK1 pathway to promote autophagy

**Fig 9:**
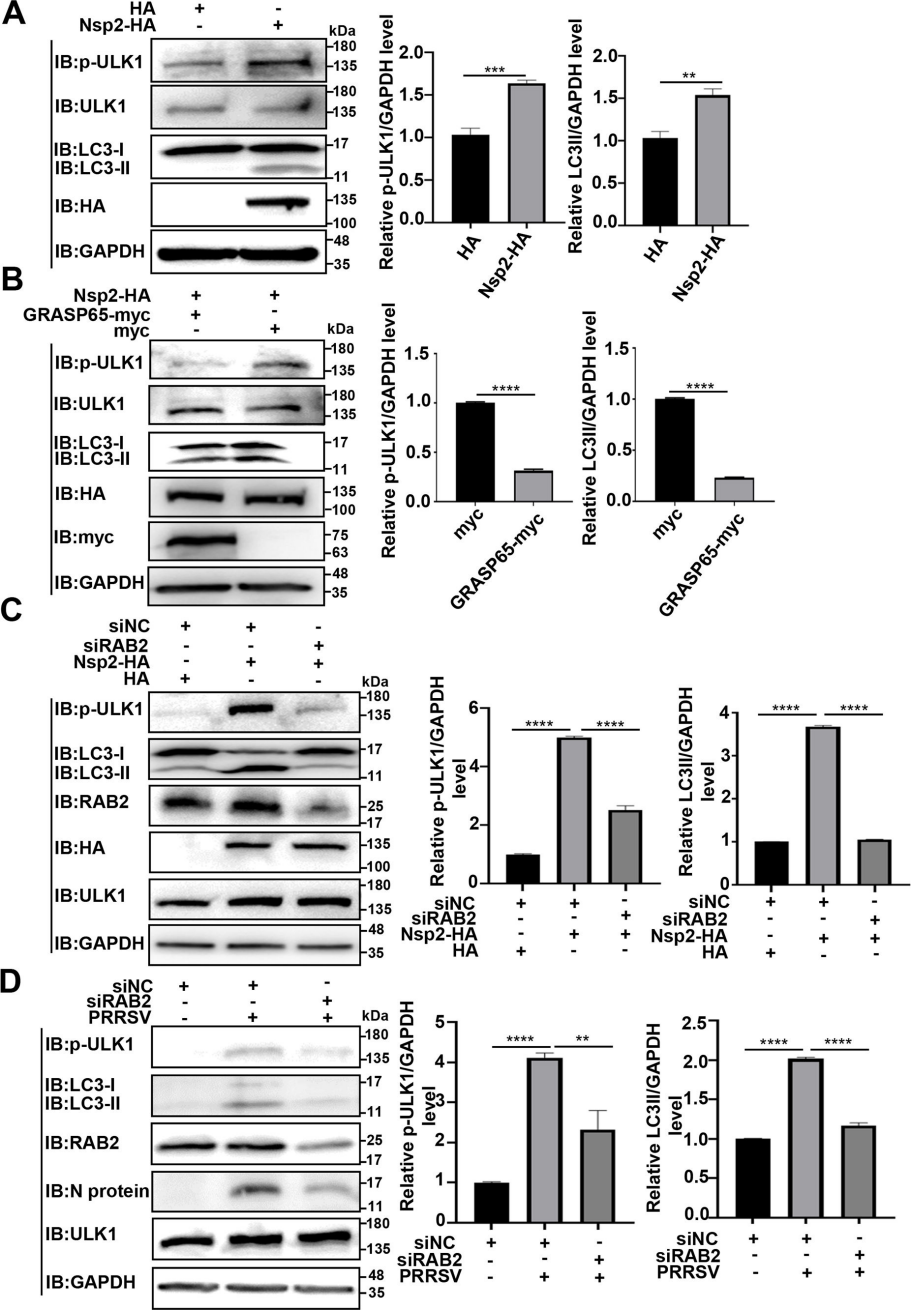
PRRSV Nsp2 induces GA fragmentation to promote autophagy by activating the RAB2-ULK1 pathway. (**A**) HEK-293T cells were transfected with the plasmid encoding Nsp2-HA or HA-tagged empty vector for 36 h. The cell lysates were collected for IB. (**B**) HEK-293T cells were co-transfected with the plasmids encoding Nsp2-HA and GRASP65-myc or myc-tagged empty vector. At 36 h post-transfection, the cell lysates were collected for IB. (**C and D**) HEK-293T or MARC-145 cells were transfected with siRAB2 or siNC. At 24 h post-transfection, the HEK-293T cells were transfected with the plasmid encoding Nsp2-HA or HA-tagged empty vector, and the MARC-145 cells were infected with PRRSV at an MOI of 1. The cell lysates were collected for IB. Data represent means ± SEM from three independent experiments. Statistical analysis was carried out using Student’s *t* test. **P < 0.01, ***P < 0.001, and ****P < 0.0001.

Based on the above results, we continued to examine whether PRRSV Nsp2-induced GA fragmentation activated the RAB2-ULK1 pathway to promote autophagy. As shown in Fig. 9A, Nsp2-HA overexpression enhanced ULK1 phosphorylation (p-ULK1) and increased LC3-II production to promote autophagy. As expected, overexpression of GRASP65-myc significantly inhibited phosphorylation of ULK1 and correspondingly decreased the level of LC3-II (Fig. 9B). To further demonstrate the role of RAB2 in the phosphorylation of ULK1 and promotion of autophagy by Nsp2, we transfected HEK-293T cells with small interference RNA (siRNA) targeting RAB2 (siRAB2) with siRNA-negative control (siNC) as a control. The results showed that knockdown of RAB2 attenuated Nsp2-HA-enhanced ULK1 phosphorylation and LC3-II production (Fig. 9C). In the PRRSV-infected cells, ULK1 phosphorylation and LC3-II production were decreased when endogenous RAB2 was knocked down (Fig. 9D). Taken together, these data demonstrate that Nsp2 induces GA fragmentation to promote autophagy by activating the RAB2-ULK1 pathway.

### PRRSV triggers GA fragmentation-mediated autophagy to facilitate viral self-replication

**Fig 10:**
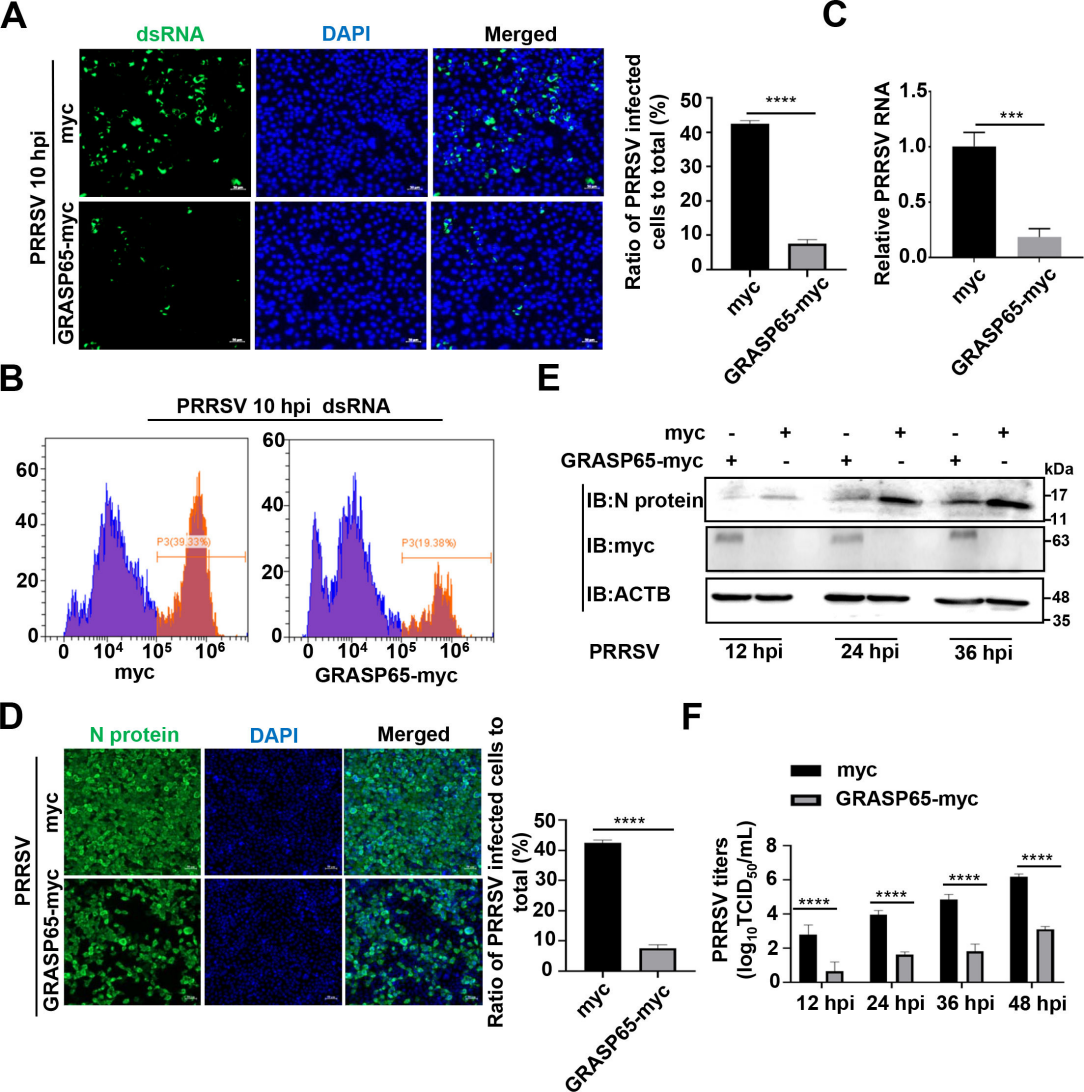
PRRSV triggers GA fragmentation-mediated autophagy to facilitate viral self-replication. (**A through C**) MARC-145 cells were transfected with the plasmid encoding GRASP65-myc or myc-tagged empty vector. At 24 h post-transfection, the MARC-145 cells were infected with PRRSV at an MOI of 5 for 10 h. (**A**) The fluorescent signal of PRRSV dsRNA abundance was detected using IFA with a mouse anti-dsRNA mAb. Nuclei were stained with DAPI. Scale bars = 50 µm. (**B**) PRRSV dsRNA abundance was detected using FCM with the mouse anti-dsRNA mAb. (**C**) PRRSV RNA abundance was detected using RT-qPCR. (**D**) MARC-145 cells were transfected with the plasmid encoding GRASP65-myc or myc-tagged empty vector for 24 h, and PRRSV infectivity was detected using IFA. IFA images were taken by confocal microscopy, and the numbers of PRRSV N protein-expressed and DAPI-stained cells were counted using ImageJ software. The infection rate was expressed as PRRSV N protein-expressed cells/DAPI-stained cells (scale bars = 50 µm). (**E and F**) MARC-145 cells were transfected with the plasmid encoding GRASP65-myc or myc-tagged empty vector. At 24 h post-transfection, the MARC-145 cells were infected with PRRSV at an MOI of 1 or mock infected for indicated time periods (12, 24, 36 h, or 48 h). (**E**) PRRSV N protein abundance was detected using IB. (**F**) PRRSV titers were measured by assessing TCID_50_. Data represent means ± SEM from three independent experiments. Statistical analysis was carried out using Student’s *t* test. ***P < 0.001 and ****P < 0.0001.

Previous studies have shown that PRRSV infection triggers autophagy to facilitate viral replication (15–17, 19, 20, 46). As PRRSV Nsp2 induced GA fragmentation to promote autophagy, we firstly investigated the effect of autophagy promoted by Nsp2-induced GA fragmentation on viral replication. We attempted to prepare a mutant PRRSV incapable of inducing GA fragmentation by mutagenesis of the residues responsible for Nsp2 cysteine protease activity (referred as in Fig. 6) in an infectious cDNA clone. Unfortunately, the virus could not be rescued (data not shown), suggesting that these residues are crucial for PRRSV replication, consistent with previous studies (39, 47). In the case that overexpression of GRASP65 inhibited PRRSV/Nsp2-induced GA fragmentation (Fig. 5), we assessed PRRSV RNA replication by detecting the intermediate of PRRSV RNA synthesis, double-stranded RNA (dsRNA), in the GRASP65-myc-overexpressed-MARC-145 cells via indirect immunofluorescence assay (IFA) and flow cytometry (FCM) at 10 hpi during the first viral life cycle (48). The results showed significant reductions in dsRNA abundance as indicated by ~75% reduction detected by IFA (Fig. 10A) and a ~70% reduction detected by FCM (Fig. 10B), respectively. RT-qPCR also showed a markedly lowered abundance of PRRSV RNA in the GRASP65-myc-overexpressed cells at 10 hpi (~75% reduction; Fig. 10C). Moreover, overexpression of GRASP65-myc significantly decreased PRRSV infectivity at 24 hpi (~80% detected by IFA; Fig. 10D). We further tested PRRSV N protein levels and titers at different time points in the GRASP65-myc-overexpressed cells. There were significant reductions in the levels of PRRSV N protein (Fig. 10E) and at least 100-fold reductions in viral titers (>2 log_10_TCID_50_ mL^−1^; Fig. 10F) via assessing 50% tissue culture infected dose (TCID_50_). These data show that PRRSV triggers GA fragmentation-mediated autophagy to promote viral self-replication.

## DISCUSSION

GA fragmentation is crucial for its functional role (49). The reversible GA fragmentation occurs in the initial stages of cell migration, division, and apoptosis, ensuring completion of various biological activities (50). The irreversible GA fragmentation is a significant factor to the onset of neurodegenerative diseases, such as Alzheimer disease, Parkinson disease, and amyotrophic lateral sclerosis (51). Additionally, sustained fragmentation of GA occurs in lung cancer, gastric cancer, and breast cancer, exacerbating cancer progression (52). An increasing number of experiments show that virus-triggered GA fragmentation functions in various stages of viral replication cycle. Hepatitis C virus and severe acute respiratory syndrome coronavirus 2 (SARS-CoV-2) promote self-replication by inducing GA fragmentation (53, 54). Influenza A virus induces GA fragmentation to modulate vesicular transport, which facilitates intracellular transport of virus particles (55). Foot-and-mouth disease virus induces GA fragmentation and blocks intra-GA transport, suppressing innate immunity (56). In the present study, we reveal that PRRSV induces GA fragmentation to promote autophagy, which facilitates viral replication for the first time.

Firstly, we motioned that different PRRSV strains induced GA fragmentation in different host cells (Fig. 1; Fig. S1). We subsequently identified that PRRSV Nsp2 significantly induced GA fragmentation (Fig. 2; Fig. S2). PRRSV Nsp2 has been previously reported to cleave viral polyproteins to produce Nsps, participate in the assembly of viral replication complexes, and inhibit innate immunity (47, 57–59). Therefore, we have extended the novel function of Nsp2 in GA fragmentation during PRRSV infection.

Through LC-MS/MS screening and other approaches, we identified that Nsp2 interacted with GRASP65 (Fig. 3 and 4; Fig. S3). The maintenance of the GA stack structure relies on Golgins, GRASPs, microtubules, microfilaments, and other related proteins (60). When there are changes in the protein levels, post-translational modifications (for example, phosphorylation), interactions, and/or subcellular localization of these proteins, the GA stack structure undergoes fragmentation (27). The protein and phosphorylation levels of GRASP65 is important in the maintenance of GA morphology (32, 61–63). Phosphorylation of GRASP65 is previously reported to affect GA morphology during human cytomegalovirus infection (61). Interestingly, we found that PRRSV Nsp2 degraded GRASP65, which resulted in GA fragmentation in the current study (Fig. 5). In addition to GRASP65, GRASP55 has been reported to play a critical role in maintaining GA morphology and its degradation is involved in SARS-CoV-2-induced GA fragmentation (64–66). Interestingly, the protein level of GRASP55 was stable during PRRSV infection (Fig. S5A). Furthermore, PRRSV Nsp2 neither interacted with GRASP55 nor influenced GRASP55 abundance (Fig. S5B and C). These results indicated that GRASP55 was not involved in Nsp2-induced GA fragmentation during PRRSV infection. It will be interesting to address this discrepancy with previous studies in the future.

The autolysosomal pathway and the ubiquitin-proteasome system are the two main intracellular protein degradation ways in eukaryotic cells (35, 36). However, we were unable to reverse the degradation of GRASP65 and GA fragmentation by Nsp2 with the addition of the corresponding inhibitors (Fig. 6). Here, we validated that GRASP65 was degraded by Nsp2 dependent on its cysteine protease activity (Fig. 6). Thereby, we discover GRASP65 as a new cleavage target of Nsp2.

Next, we determined that PRRSV Nsp2 induced GA fragmentation to promote autophagy (Fig. 7). There exists a close relationship between GA fragmentation and autophagy (28). We demonstrated that autophagy was promoted during GA fragmentation whereas it was correspondingly suppressed when GA fragmentation was inhibited by overexpression of GRASP65 (Fig. 7). We further elucidated that PRRSV Nsp2-induced GA fragmentation activated the RAB2-ULK1 pathway to promote autophagy (Fig. 8 and 9). In detail, we found that upon GA fragmentation, RAB2 was disassociated from GM130, while it interacted with ULK1 (Fig. 8), which enhanced ULK1 phosphorylation and promoted autophagy (Fig. 9 and Fig. S4). Consistently, we found that knockdown of GRASP65 attenuated the association of RAB2 with GM130, whereas it enhanced its interaction with ULK1, thereby promoting autophagy (Fig. S6).

In fact, several other GA-resident proteins are shown to play an important role in autophagy, such as immunity-related GTPase M (67) and GABARAP (68). However, we detected that knockdown of these two proteins did not affect autophagy in the Nsp2-overexpressed or PRRSV-infected cells (data not shown). This discrepancy is another fascinating issue to be addressed. In addition to the RAB2-ULK1 pathway, the RAB1a-ULK1 pathway has been revealed to take effect in PRRSV-induced autophagy (46), indicating the importance of RABs in autophagic processes. Previous studies have uncovered that PRRSV Nsp2 interacts with the cellular protein 14-3-3ε and induces the formation of aggresomes, which promotes autophagy (20) as well as induces endoplasmic reticulum stress to trigger autophagy (19). As a consequence, we provide a distinctive mechanism of Nsp2-induced autophagy in this work.

Finally, we demonstrated that PRRSV triggered GA fragmentation-mediated autophagy to facilitate viral self-replication and overexpression of GRASP65 inhibited PRRSV replication when antagonizing GA fragmentation (Fig. 10). Earlier reports show that viruses induce GA fragmentation and utilize GA membrane fragments and vesicles for assembly of replication complexes (53, 54, 69). Consequently, we have provided a different manner of GA fragmentation for promoting virus replication via autophagy. PRRSV has been reported to induce double-membrane vesicles (DMVs) by appropriating membranes from host cell organelles for viral replication (70). Therefore, it is actually intriguing to investigate the involvement of GA fragmentation in the formation of DMVs and we will address it in our future research.

**Fig 11:**
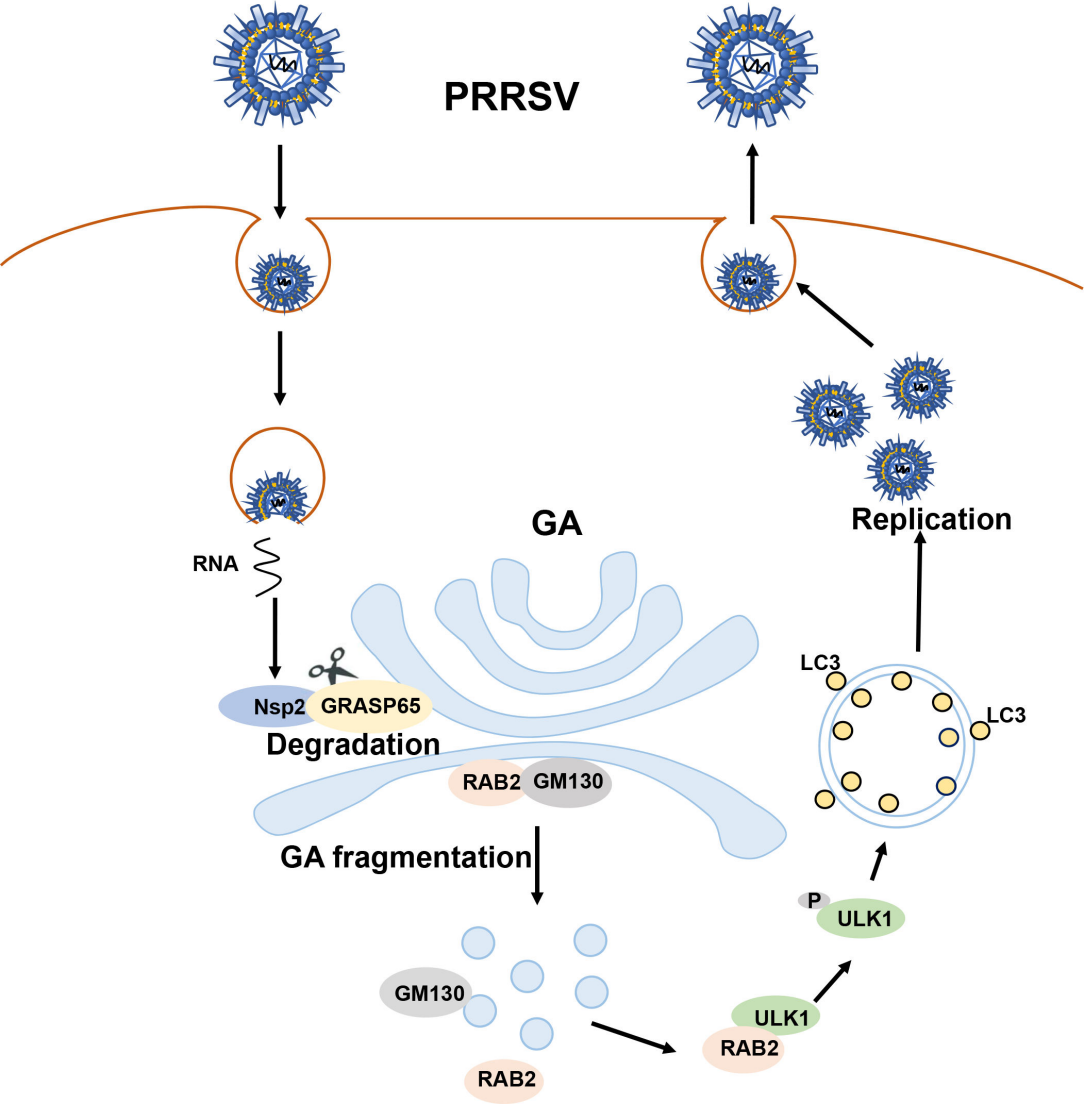
The schematic model depicting that PRRSV triggers GA fragmentation-mediated autophagy to facilitate viral self-replication. Mechanistically, PRRSV Nsp2 is identified to interact with and degrade GRASP65, resulting in GA fragmentation. Upon GA fragmentation, GA-resident RAB2 is disassociated from GM130. The dissociated RAB2 interacts with ULK1 and enhances ULK1 phosphorylation, promoting autophagy and viral replication.

Based on these results, we propose a model to describe that PRRSV triggers GA fragmentation-mediated autophagy to facilitate viral self-replication (Fig. 11). During PRRSV infection, Nsp2 induces GA fragmentation by degrading GRASP65, which leads to the disassociation of the GA-resident RAB2 from GM130. The dissociated RAB2 interacts with ULK1 and enhances ULK1 phosphorylation, promoting autophagy and viral replication.

In conclusion, our study reveals that PRRSV promotes autophagy via inducing GA fragmentation to facilitate viral infection. These findings expand our understanding of PRRSV pathogenesis and autophagy. More importantly, given its importance in multiple viral infections, GA fragmentation can be exploited as a broad-spectrum antiviral target.

## MATERIALS AND METHODS

### Cells and viruses

MARC-145, HEK-293T, HeLa, and CRL-2843-CD163 cell lines were stored in our laboratory (71). MARC-145 and HEK-293T cells were maintained in DMEM (Solarbio, Cat. No. 12100) supplemented with 10% heat-inactivated FBS (Gibco, Cat. No. 10270–106), and 100 units mL^−1^ penicillin and 100 mg mL^−1^ streptomycin sulfate (Sangon, Cat. No. B540732) at 37°C in 5% CO_2_. HeLa cells were routinely maintained in modified Eagle medium (iCell Bioscience Inc., Cat. No. 138–0012) supplemented with 10% heat-inactivated FBS and 100 units mL^−1^ penicillin and 100 mg mL^−1^ streptomycin sulfate at 37°C in 5% CO_2_. CRL-2843-CD163 cells were maintained in Roswell Park Memorial Institute 1640 medium (Solarbio, Cat. No. 31800) supplemented with 10% FBS and 100 units mL^−1^ penicillin and 100 mg mL^−1^ streptomycin sulfate at 37°C in 5% CO_2_.

HP-PRRSV strain HN07-1 (GenBank: KX766378.1) and NADC30-like PRRSV strain HNhx (GenBank: KX766379) were previously isolated by our laboratory (72, 73). Low-pathogenic PRRSV strain BJ-4 (GenBank: AF331831) was kindly provided by Professor Hanchun Yang of China Agricultural University. The HP-PRRSV strain HN07-1 was utilized in the current study unless otherwise stated.

### Antibodies

Anti-rabbit GRASP65 monoclonal antibody (mAb, Cat. No. ab249824), Alexa Fluor 555-goat anti-rabbit IgG polyclonal antibodies (pAbs, Cat. No. ab150078), Alexa Fluor 488-goat anti-rabbit IgG pAbs (Cat. No. ab150077), Alexa Fluor 647-goat anti-rabbit IgG pAbs (Cat. No. ab150115), horseradish peroxidase (HRP)-labeled goat anti-rabbit IgG pAbs (Cat. No. ab6721), and HRP-labeled goat anti-mouse IgG pAbs (Cat. No. ab6789) were purchased from Abcam. Rabbit anti-GM130 mAb (Cat. No. 12480), rabbit anti-ULK1 mAb (Cat. No. 8054), mouse anti-GST mAb (Cat. No. 2624), mouse anti-His mAb (Cat. No. 9991), rabbit anti-Flag mAb (Cat. No. 14793), mouse anti-myc mAb (Cat. No. 2276 s), rabbit anti-HA mAb (Cat. No. 3724), rabbit anti-LC3B mAb (Cat. No. 2775), and rabbit anti-phospho-ULK1 mAb (Cat. No. 14202) were purchased from Cell Signaling Technology. Rabbit anti-PRRSV N pAbs (Cat. No. GTX129270) for IB was purchased from GeneTex. Rabbit anti-GRASP55 pAbs (Cat. No. 10598–1-AP-1), rabbit anti-ACTB mAb (Cat. No. 81115–1-RR), and mouse anti-glyceraldehyde-3-phosphate dehydrogenase (GAPDH) mAb (Cat. No. 60004–1-Ig) were purchased from Proteintech. Mouse anti-dsRNA mAb (J2, Cat. No. 10010200) was purchased from Scicons. Mouse anti-RAB2A mAb (Cat. No. sc-515612) was purchased from Santa Cruz Biotechnology. Mouse anti-IgG antibody (Cat. No. A7028) was purchased from Beyotime. Mouse anti-PRRSV N mAb for IFA was prepared by our laboratory (74). Anti-PRRSV Nsp2 mAb for IFA was kindly provided by Professor Hanchun Yang of China Agricultural University.

### Preparation of pAbs targeting PRRSV Nsp2 for IB

The cDNA of PLP2-His were synthesized and cloned into the pET-28a plasmid by GENEWIZ. Recombinant PLP2-His was expressed in *Escherichia coli* BL21-competent cells (TransGen, Cat. No. CD601-02) and purified by Ni-NTA agarose (Sigma-Aldrich, Cat. No. R90115). To prepare mouse pAbs against Nsp2, 40 µg of the purified PLP2-His was emulsified in Freund’s complete adjuvant or Freund’s incomplete adjuvant (Sigma, Cat. No. F5881 and F5506) and then injected into 4–6-week-old female BALB/c mice. The immunization was repeated three times every 2 weeks. After three immunizations, the blood was collected from the eye and the serum was used as the pAbs against Nsp2 for IB. The experimental procedure was permitted by the Ethical and Animal Welfare Committee of Key Laboratory of Animal Immunology of the Ministry of Agriculture of China (Approval No. LLSC4102223). There were no endangered or protected species involved in the research.

### Plasmid constructs and transfection

The cDNA of each protein, Nsp2 deletion mutants, and Nsp2-mutant-HA from PRRSV strain HN07-1 as well as Nsp2 from PRRSV strains BJ-4 and HNhx were synthesized and cloned into the pCAGGS-HA plasmid. The cDNA of GRASP65 (GeneBank: AJ409349.1) was constructed into the pcDNA3.1-myc/his_A, pGEX-6p-1, and pEGFP-C1 plasmids, respectively. The cDNA of GRASP55 (GeneBank: NM_015530.5) was constructed into the pEGFP-C1 plasmid. The cDNA of RAB2 (GeneBank: M28213.1) was constructed into the pcDNA3.1-myc/his_A and pEGFP-C1 plasmids, respectively. All the plasmids were constructed by GENEWIZ. The pCMV-ULK1−3×Flag-Neo plasmid (Cat. No. P39375) was purchased from MiaoLing Biology.

For the transfection, the plasmids were transfected with Lipofectamine 2000 (Thermo Fisher Scientific, Cat. No. 2066194) or TransIntro PL Transfection Reagent (TransGen Biotech, Cat. No. FT301-01) according to the manufacturer’s instructions. Unless otherwise stated, 12-well cell culture plates were transfected with 1.5 µg of each plasmid per well, and 6-well cell culture plates were transfected with 2.5 µg of each plasmid per well.

### IFA and confocal microscopy

MARC-145, HeLa, or CRL-2843-CD163 cells were washed with cold phosphate buffered solution (PBS; Solarbio, Cat. No. P1010), fixed with 4% paraformaldehyde (Solarbio, Cat. No. P1110) for 15 min, and permeabilized with 0.1% Triton X-100 (Solarbio, Cat. No. T8200) at room temperature (RT) for 10 min. After three rinses with cold PBS, the cells were incubated with PBS containing 5% bovine serum albumin at RT for 1 h, followed by incubation with the primary antibodies at 4°C overnight. The cells were washed three times with PBS and incubated with the appropriate fluorescent secondary antibodies for 1 h. Cell nuclei were stained with 4′,6-diamidino-2-phenylindole (DAPI, Solarbio, Cat. No. C0060) for an additional 5 min. Finally, the fluorescent images were observed via a fluorescence microscope (LSM800, Carl Zeiss AG, Oberkochen, Germany) with a confocal laser scanning set up (20×, 40×, or 63×) and were representative as a single slice of a stack from three independent experiments (75). The co-localization analyses were performed using the JaCoP plugin in ImageJ software according to previous research guidelines (76–78). Pearson’s correlation coefficient (>0.5) describes the correlation of the intensity distribution between channels.

### TEM

HeLa cells were inoculated in 6-well plates transfected with the target plasmids for 36 h, or MARC-145 cells were infected with PRRSV strain HN07-1 for 12 h. The cells were collected and fixed using 2.5% glutaraldehyde fixative (Servicebio, Cat. No. G1102) at RT for 30 min. The fixed cells were dehydrated with a graded series of ethanol and finally embedded in pure LR White resin at 4°C (HaideBio, Cat. No. 14381-UC) using BEEM capsules (Electron Microscopy Sciences, Cat. No. 69920–00). After polymerization with a low-temperature UV polymerizer (UVCC2515; Electron Microscopy, Beijing, China), the resin blocks were sliced into 70- to 80-nm thin slices on an ultramicrotome (UC7; Leica, Wetzlar, Germany), and the ultrathin sections were then fished out onto the 150-mesh nickel grids with a Formvar film (Head Biotechnology Co., Cat. No. FCF200-Cu-50). Finally, the samples were observed at 80 kV using TEM (Ht7800/Ht7700; Hitachi, Tokyo, Japan). Representative images were shown.

### HA-IP/MS

HEK-293T cells were transfected with the plasmids expressing Nsp2-HA and HA-tagged empty vector using TransIntro PL Transfection Reagent. At 36 h post-transfection, the cells were lysed on ice with Western blotting (WB)/IP lysis buffer (Solarbio, Cat. No. R0100). The cell lysates were then centrifuged at 12,000 × *g* for 10 min. Anti-HA magnetic beads (MedChemExpress, Cat. No. HY-K0201) were incubated with the supernatants. After incubation at 4°C overnight, the beads were washed five times with PBS. The associated proteins were analyzed by 12.5% SDS-PAGE (Epizyme, Cat. No. PG113), and the protein bands in the gel were stained with a fast silver stain kit (Beyotime, Cat. No. P0017S). The indicated protein bands were cut and applied to LC-MS/MS by Lumingbio (Shanghai, China). The top-ranked peptide matches were taken into consideration for protein identification. Representative images were shown.

### IB

The cells were lysed on ice with WB/IP lysis buffer containing a protease inhibitor cocktail (Roche, Cat. No. 04693116001). Protein samples were normalized to equal amounts of GAPDH/ACTB, separated by SDS-PAGE, and transferred onto 0.22 µm polyvinylidene fluoride membranes (Merck, Cat. No. ISEQ00010), which were blocked in 5% skimmed milk at RT for 2 h. The membranes were incubated with the primary antibodies at 4°C overnight and then incubated with the corresponding secondary antibodies (conjugated with HRP) at RT for 1 h. After washing, IB results were visualized with enhanced chemiluminescence reagents (NCM Biotechnology, Cat. No. P10300) and imaged using a chemiluminescence imaging system (Fusion FX7; VILBER, Paris, France). Representative images were shown.

### Co-IP

HEK-293T cells were transfected with the indicated plasmids for 36 h and lysed on ice with WB/IP lysis buffer containing a protease inhibitor cocktail. The cell lysates were centrifuged and incubated with anti-HA magnetic beads, anti-myc magnetic beads (MedChemExpress, Cat. No. HY-K0206-5), or anti-Flag magnetic beads (MedChemExpress, Cat. No. HY-K0207) at 4°C overnight. The precipitated immune complexes were collected with a magnetic holder and washed with PBS. Finally, the complexes were eluted with a 2× SDS loading buffer (TaKaRa, Cat. No. 9173) and subjected to IB with the indicated antibodies.

### IP

The cells were transfected with the target plasmids for 36 h and lysed with WB/IP lysis buffer containing the protease inhibitor cocktail for 20 min and centrifuged at 12,000 × *g* for 10 min. The supernatant was assayed according to the manufacturer’s instructions for the protein A/G magnetic beads (Invitrogen, Cat. No. 10004D). In brief, the supernatant was incubated with mouse anti-Nsp2 pAbs or mouse Isotype IgG (Beyotime, Cat. No. A7028) at RT for 2 h. The mixture was then added to the samples and incubated at 4°C overnight. The precipitated immune complexes were collected with a magnetic holder and washed with PBS. Finally, the complexes were eluted with a 2× SDS loading buffer and subjected to IB with the indicated antibodies.

### GST pulldown

The target proteins were expressed in *Escherichia coli* BL21 competent cells and purified by GST magnetic beads (Beaver, Cat. No. 70601). GST magnetic beads were incubated with the purified GRASP65-GST at RT for 2 h and then purified PLP2-His overnight at 4°C. After washing six times with PBS, the samples were eluted and assayed by IB.

### RT-qPCR

Total RNAs were extracted using a RNAiso Plus reagent (TaKaRa, Cat. No. 9108) and reversely transcribed into cDNAs with a PrimeScript RT master mix kit (TaKaRa, Cat. No. RR047A). The cDNAs from different samples were amplified by PCR using a universal SYBR green master (Roche, Cat. No. 04913914001) on LightCycler480 II (Roche, Basel, Switzerland). The relative mRNA abundance was normalized to the expression of GAPDH and evaluated by the 2^-△△CT^ method (79). The primer sequences used for RT-qPCR were listed in Table S1.

### Inhibitor treatments

HEK-293T or HeLa cells were transfected with the plasmid encoding Nsp2-HA for 6 h. Next, the cells were treated with dimethyl sulfoxide (DMSO, Sigma-Aldrich, Cat. No. 276855), 3-MA (Sigma-Aldrich, Cat. No. M9281), or MG132 (Sigma-Aldrich, Cat. No. M7449) and the samples were collected after 24 h. The cell viability assay was performed according to our previous study (data not shown) (80).

### RNA interference

All siRNAs and siNC were designed and synthesized by GenePharma (Shanghai, China). The cells were transfected with the indicated siRNAs using Lipofectamine RNAiMAX (Thermo Fisher Scientific, Cat. No. 13778057) according to the manufacturer’s instructions. The siRNA sequences were listed in Table S2. The cell viability assay was performed according to our previous study (data not shown) (80).

### FCM

The infected MARC-145 cells were digested with 0.25% trypsin-EDTA solution (Solarbio, T1320), collected by centrifugation, and re-suspended in PBS. The cells were fixed with 4% paraformaldehyde, permeabilized with 0.1% Triton X-100, and then blocked with 5% bovine serum albumin. The cells were incubated with or without mouse anti-dsRNA mAb at RT for 2 h and Alexa Fluor 647-goat anti-mouse IgG pAbs for 1 h. Finally, the washed cells were resuspended in PBS and analyzed by a flow cytometer (CytoFLEX; Beckman Coulter, Brea, USA). Representative images were shown.

### PRRSV titration assay

The infected cells were subjected to three freeze-thaw cycles and centrifuged at 5,000 × *g* for 10 min to remove cellular debris and obtain PRRSV supernatants. MARC-145 cells were seeded in 96-well cell culture plates and inoculated with serially diluted PRRSV supernatants (10^−1^–10^−8^-fold) at 37°C for 1 h. The excess virus inoculum was removed by washing with PBS. Each well was added with 100 µL maintenance DMEM containing 2% FBS and the cells were cultured for 3 to 5 d. The TCID_50_ value was calculated according to the Reed-Muench method (81).

### Statistical analysis

Each experiment consisted of three replicates and was independently repeated at least three times. The data represent means ±  standard error of the mean from three independent experiments. All data and calculations were analyzed in Prism 8.0 software (San Diego, USA) using unpaired two-tailed Student’s *t* test. Statistical significance was represented by asterisks [ns, not significant (*P* > 0.05); *P < 0.05, **P < 0.01, ***P < 0.001, and ****P < 0.0001).

## SUPPLEMENTAL MATERIAL

Fig. S1 jvi.01842-23-s0001.tif PRRSV infection induces GA fragmentation.

Fig. S2 jvi.01842-23-s0002.tif PRRSV strains BJ-4 and HNhx Nsp2 induce GA fragmentation.

Fig. S3 jvi.01842-23-s0003.tif PRRSV Nsp2 interacts with GRASP65.

Fig. S4 jvi.01842-23-s0004.tif GRASP65 overexpression counteracts Nsp2-induced GA fragmentation to restore the association of RAB2 with GM130 and attenuate its interaction with ULK1.

Fig. S5 jvi.01842-23-s0005.tif PRRSV Nsp2 neither interacts with nor changes the protein level of GRASP55 during infection.

Fig. S6 jvi.01842-23-s0006.tif Knockdown of GRASP65 attenuates the association of RAB2 with GM130 and enhances its interaction with ULK1 to promote autophagy.

Supplemental legends jvi.01842-23-s0007.docx Legends for Fig. S1 to S6.

Table S1 jvi.01842-23-s0008.docx The primers used for RT-qPCR in this study.

Table S2 jvi.01842-23-s0009.docx The sequences of siRNAs used in this study.

ASM does not own the copyrights to Supplemental Material that may be linked to, or accessed through, an article. The authors have granted ASM a non-exclusive, world-wide license to publish the Supplemental Material files. Please contact the corresponding author directly for reuse.
