# Supplementary figures and images for: Porcine reproductive and respiratory syndrome virus triggers Golgi apparatus fragmentation-mediated autophagy to facilitate viral self-replication
Source: J Virol. 2024 Jan 5;98(2):e01842-23. doi: 10.1128/jvi.01842-23 (PMC10878038; doi:10.1128/jvi.01842-23)

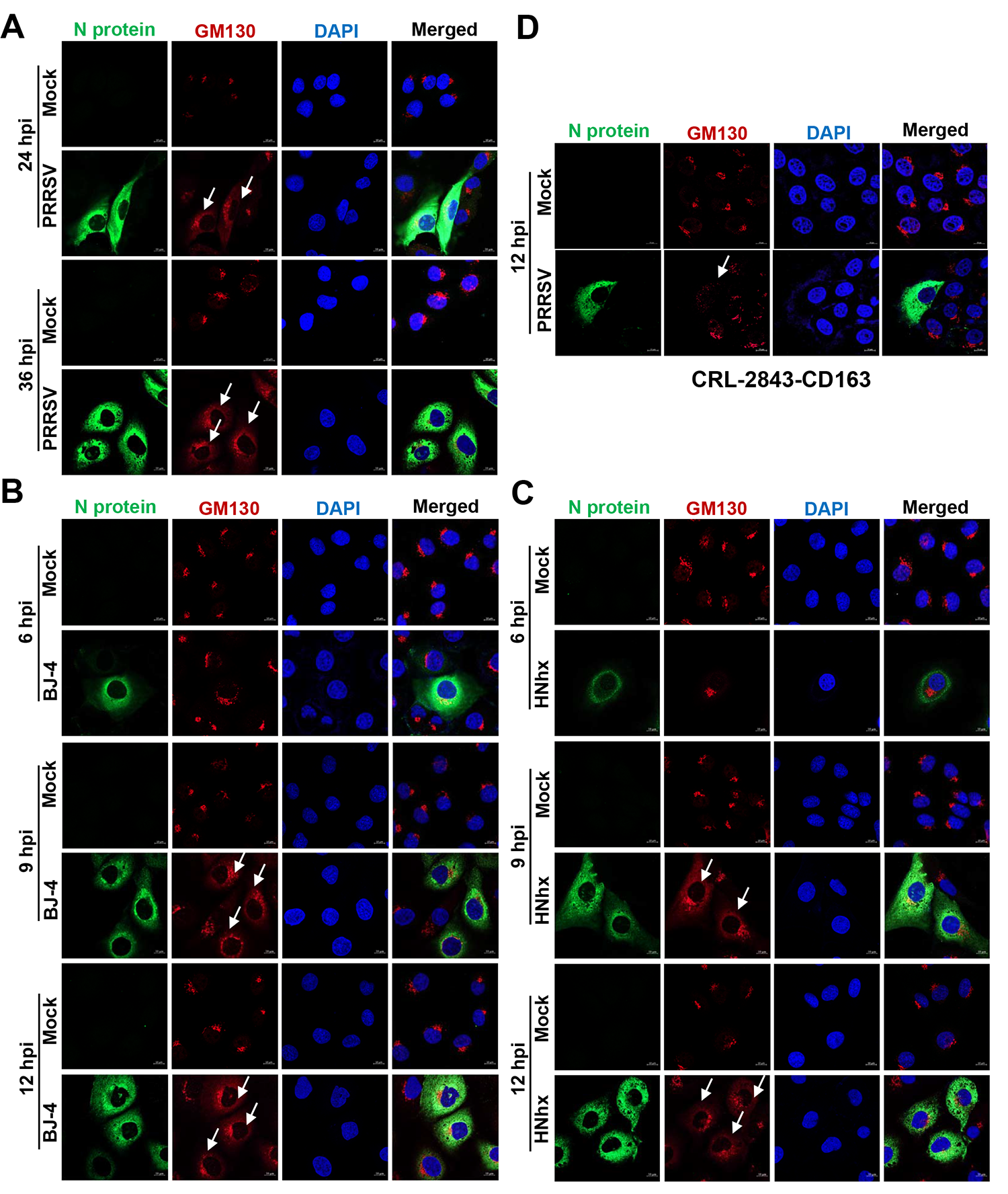

Supplement: Fig. S1 — PRRSV infection induces GA fragmentation. [file jvi.01842-23-s0001.tif]

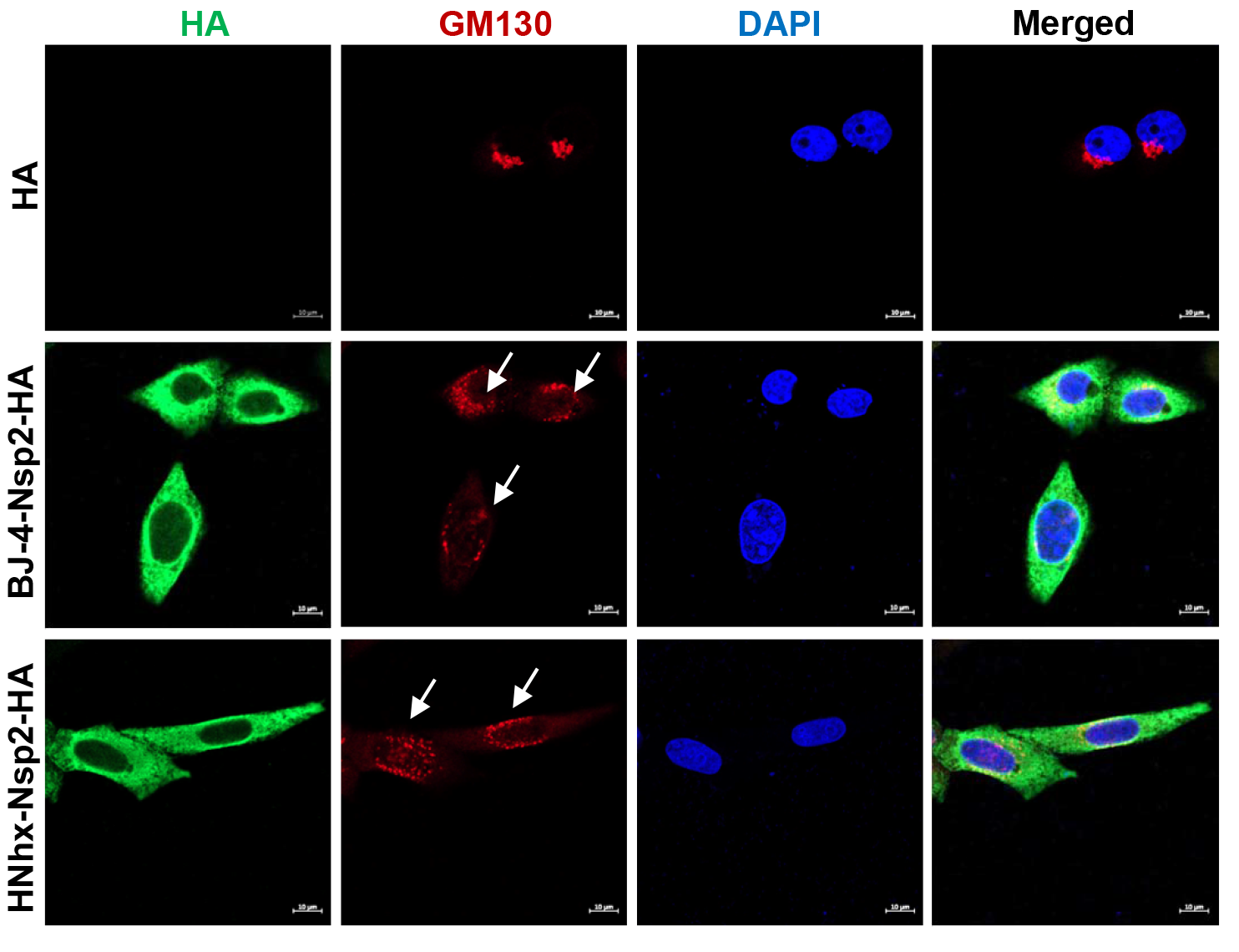

Supplement: Fig. S2 — PRRSV strains BJ-4 and HNhx Nsp2 induce GA fragmentation. [file jvi.01842-23-s0002.tif]

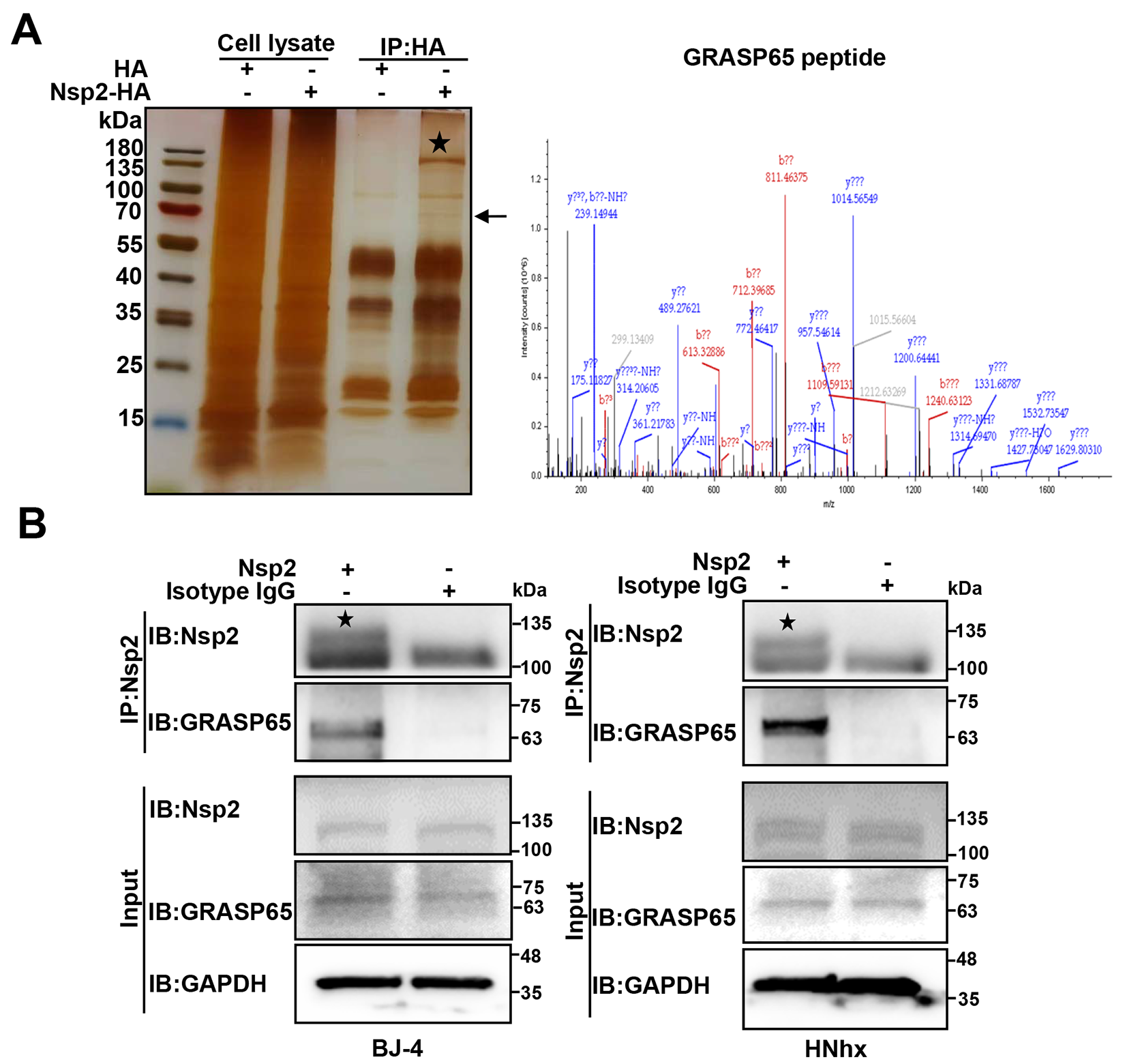

Supplement: Fig. S3 — PRRSV Nsp2 interacts with GRASP65. [file jvi.01842-23-s0003.tif]

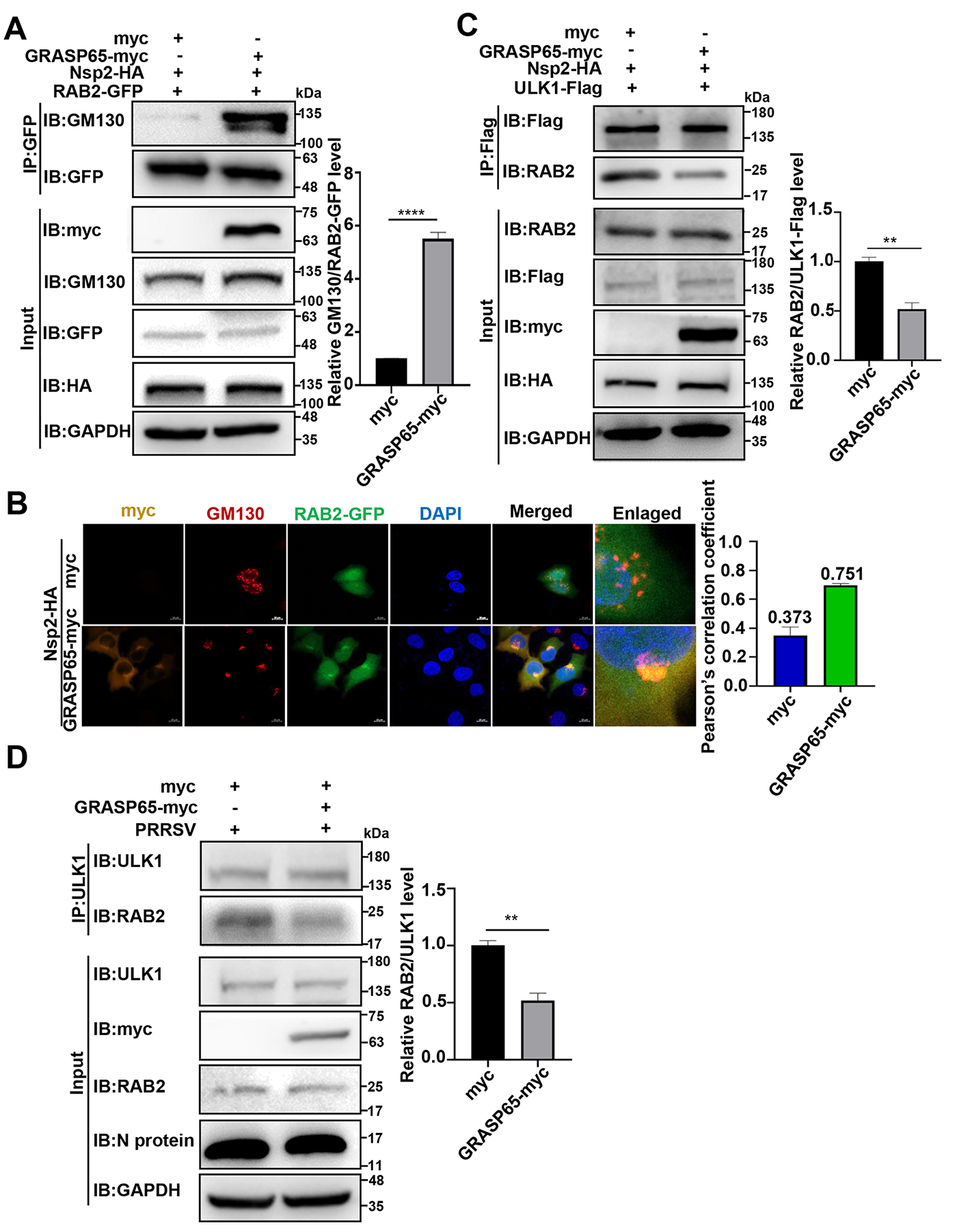

Supplement: Fig. S4 — GRASP65 overexpression counteracts Nsp2-induced GA fragmentation to restore the association of RAB2 with GM130 and attenuate its interaction with ULK1. [file jvi.01842-23-s0004.tif]

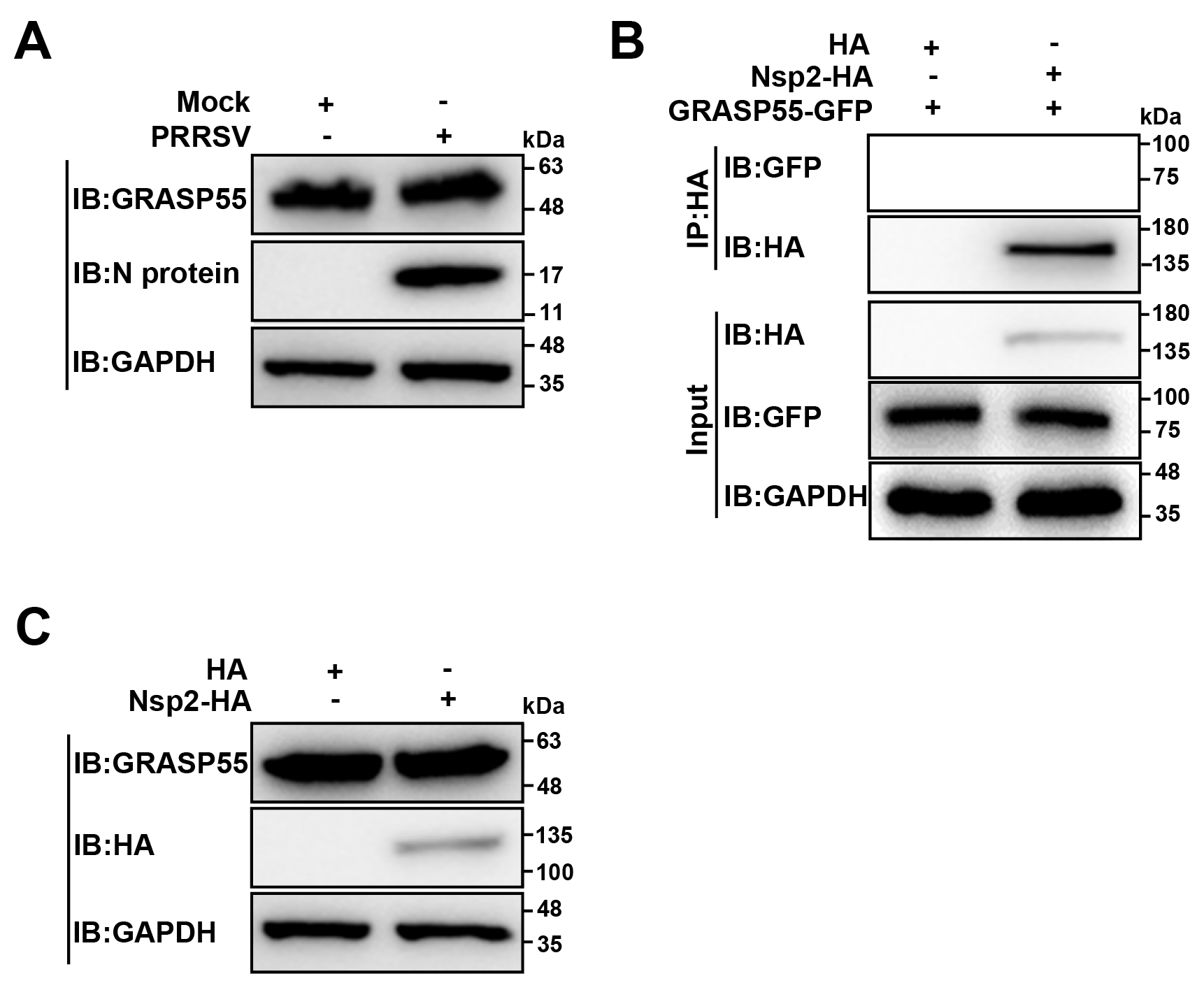

Supplement: Fig. S5 — PRRSV Nsp2 neither interacts with nor changes the protein level of GRASP55 during infection. [file jvi.01842-23-s0005.tif]

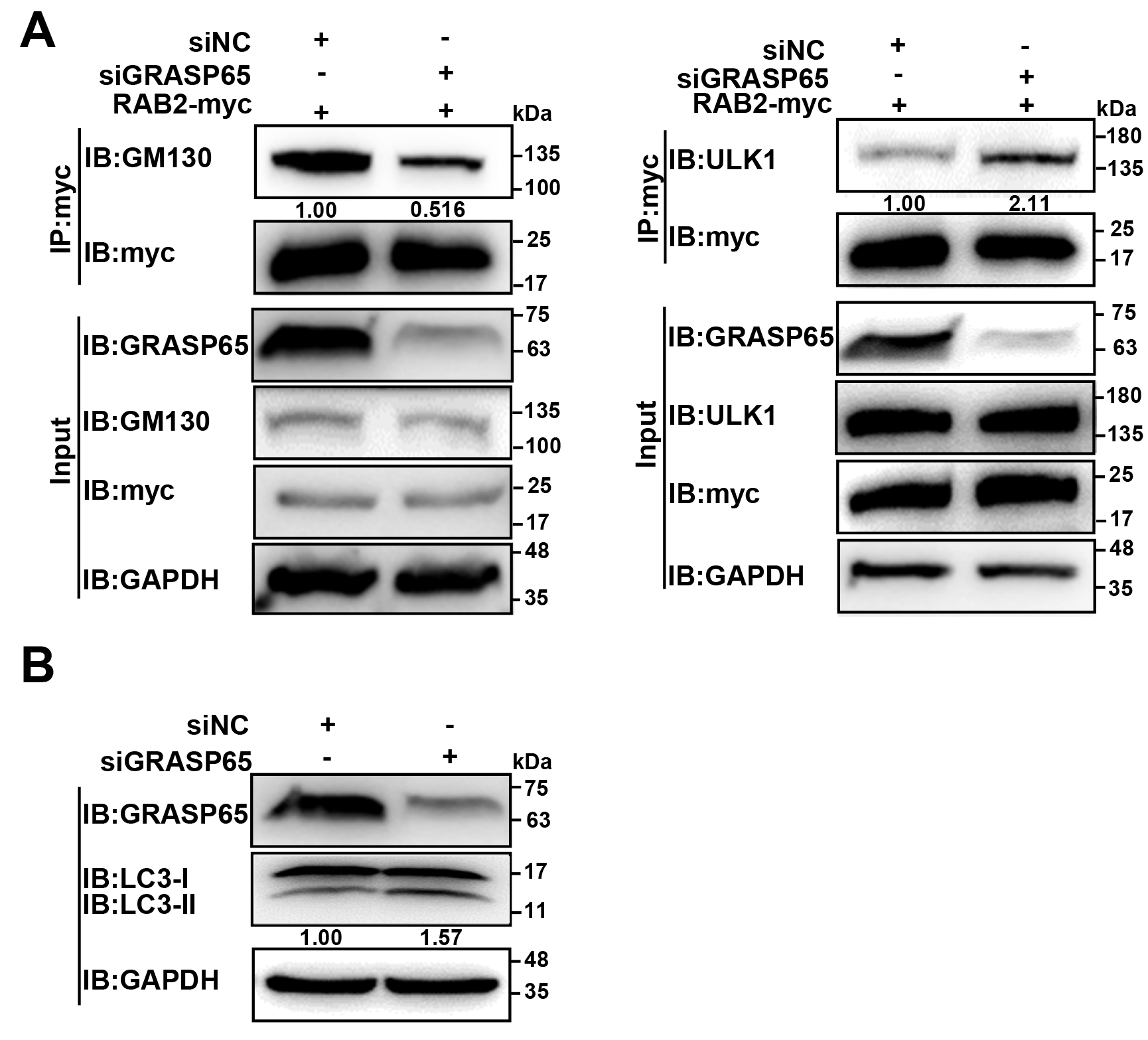

Supplement: Fig. S6 — Knockdown of GRASP65 attenuates the association of RAB2 with GM130 and enhances its interaction with ULK1 to promote autophagy. [file jvi.01842-23-s0006.tif]
