# Supplementary material for: Porcine reproductive and respiratory syndrome virus triggers Golgi apparatus fragmentation-mediated autophagy to facilitate viral self-replication
Source: J Virol. 2024 Jan 5;98(2):e01842-23. doi: 10.1128/jvi.01842-23 (PMC10878038; doi:10.1128/jvi.01842-23)
Supplement: Supplemental legends — Legends for Fig. S1 to S6. [file jvi.01842-23-s0007.docx]

**SUPPLEMENTAL FIGURE LEGENDS**

**FIG S1. PRRSV infection induces GA fragmentation.**

(**A**) The mock-infected or PRRSV strain HN07-1-infected (MOI = 1) MARC-145 cells were collected at 24 and 36 hpi. (**B** and **C**) The mock-infected or PRRSV strain BJ-4/HNhx-infected (MOI = 1) MARC-145 cells were collected at 6, 9, and 12 hpi. (**D**) The mock-infected or PRRSV strain HN07-1-infected (MOI = 1) CRL-2843-CD163 cells were collected at 12 hpi. GM130 and PRRSV N protein were visualized with the specific primary and secondary antibodies. Cell nuclei were stained with DAPI. The fluorescent signals were monitored with confocal microscopy (scale bars = 10 µm). White arrows indicated GA fragmentation.

**FIG S2.** **PRRSV strains BJ-4 and HNhx Nsp2 induce GA fragmentation.**

HeLa cells were transfected with the plasmids encoding the Nsp2-HA from PRRSV strains BJ-4 and HNhx or HA-tagged empty vector. GA morphology was assessed by confocal microscopy (scale bars = 10 µm). White arrows indicated GA fragmentation.

**FIG S3. PRRSV Nsp2 interacts with GRASP65.**

(**A**) HEK-293T cells were transfected with the plasmid expressing HA or Nsp2-HA. The proteins were immunoprecipitated in cell lysates using anti-HA antibody, separated by 12% SDS-PAGE, and stained with silver. The arrow indicated the most pronounced difference in protein bands. Asterisk marked Nsp2-HA. The panel on the right showed the tandem MS analysis of the GRASP65 peptide. (**B**) MARC-145 cells were infected with PRRSV strain BJ-4 or HNhx at 0.1 MOI for 24 h. They were then analyzed via endogenous IP using protein A/G magnetic beads pre-incubated with anti-Nsp2 pAbs, and IB with anti-Nsp2 and anti-GRASP65 antibodies. Asterisks marked PRRSV Nsp2.

**FIG S4.** **GRASP65 overexpression counteracts Nsp2-induced GA fragmentation to restore the association of RAB2 with GM130 and attenuate its interaction with ULK1.**

(**A** and **B**) HEK-293T or HeLa cells were transfected with the plasmids encoding RAB2-GFP, Nsp2-HA, and GRASP65-myc or myc-tagged empty vector for 36 h. (**A**) Co-IP was performed with anti-GFP magnetic beads and IB was conducted with the specific antibodies. (**B**) The fluorescent signals were observed with confocal microscopy. The co-localization was assessed by determination of the Pearson’s correlation coefficient (scale bars = 10 µm). (**C**) HEK-293T cells were transfected with the plasmids encoding ULK1-Flag, Nsp2-HA, and GRASP65-myc or myc-tagged empty vector for 36 h, followed by co-IP with anti-Flag magnetic beads and IB analysis with the specific antibodies. (**D**) MARC-145 cells were transfected with the plasmid encoding GRASP65-myc or myc-tagged empty vector. At 24 h post-transfection, the MARC-145 cells were infected with PRRSV at an MOI of 1 for 24 h. The cell lysates were immunoprecipitated by the ULK1 antibody pre-incubated with protein A/G magnetic beads. IB was conducted with the specific antibodies. Data represent means ± SEM from three independent experiments. Statistical analysis was carried out using the Student *t* test. **, P < 0.01.

**FIG S5. PRRSV Nsp2 neither interacts with nor changes the protein level of GRASP55 during infection.**

(**A**) MARC-145 cells were infected with PRRSV at an MOI of 1 for 24 h, followed by IB analysis with the specific antibodies. (**B**) HEK-293T cells were transfected with the plasmids encoding GRASP55-GFP, and Nsp2-HA or HA-tagged empty vector for 36 h, followed by co-IP with anti-HA magnetic beads and IB analysis with the specific antibodies. (**C**) HEK-293T cells were transfected with the plasmid encoding Nsp2-HA or HA-tagged empty vector. At 36 h post-transfection, the cell lysates were collected for IB analysis with the specific antibodies.

**FIG S6.** **Knockdown of GRASP65 attenuates the association of RAB2 with GM130 and enhances its interaction with ULK1 to promote autophagy.**

(**A**) HEK-293T cells were transfected with siGRASP65 or siNC. At 24 h post-transfection, the HEK-293T cells were transfected with the plasmid encoding RAB2-myc, followed by co-IP with anti-myc magnetic beads and IB analysis with the specific antibodies. (**B**) HEK-293T cells were transfected with siGRASP65 or siNC. At 48h post-transfection, the cell lysates were collected for IB.
