# Supplementary material for: Porcine reproductive and respiratory syndrome virus triggers Golgi apparatus fragmentation-mediated autophagy to facilitate viral self-replication
Source: J Virol. 2024 Jan 5;98(2):e01842-23. doi: 10.1128/jvi.01842-23 (PMC10878038; doi:10.1128/jvi.01842-23)
Supplement: Table S1 — The primers used for RT-qPCR in this study. [file jvi.01842-23-s0008.docx]

**TABLE S1. The primers used for RT-qPCR in this study.**

| Name | Forward sequence (5’-3’) | Reverse sequence (5’-3’) |
| --- | --- | --- |
| PRRSV-N | AAACCAGTCCAGAGGCAAGG | GCAAACTAAACTCCACAGTGTAA |
| GRASP65 | CATGGGCCTGGGCGTC | GTAGTGCCTTCAGGGTGTCA |
| GAPDH | TGACAACAGCCTCAAGATCG | GTCTTCTGGGTGGCAGTGAT |
