# Supplementary material for: Porcine reproductive and respiratory syndrome virus triggers Golgi apparatus fragmentation-mediated autophagy to facilitate viral self-replication
Source: J Virol. 2024 Jan 5;98(2):e01842-23. doi: 10.1128/jvi.01842-23 (PMC10878038; doi:10.1128/jvi.01842-23)
Supplement: Table S2 — The sequences of siRNAs used in this study. [file jvi.01842-23-s0009.docx]

**TABLE S2. The sequences of siRNAs used in this study.**

| Name | Forward sequence (5ʹ-3ʹ) | Reverse sequence (5ʹ-3ʹ) |
| --- | --- | --- |
| siRAB2 | GCCUAUCUCUUCAAGUACATT | UGUACUUGAAGAGAUAGGCTT |
| siGRASP65 | GGCUGAACAAGGAGAAUGATT | UCAUUCUCCUUGUUCAGCCTT |
| siNC | UUCUCCGAACGUGUCACGUTT | ACGUGACACGUUCGGAGAATT |
